# Neurodevelopmental signatures of narcotic and neuropsychiatric risk factors in 3D human-derived forebrain organoids

**DOI:** 10.1038/s41380-021-01189-9

**Published:** 2021-06-22

**Authors:** Michael Notaras, Aiman Lodhi, Estibaliz Barrio-Alonso, Careen Foord, Tori Rodrick, Drew Jones, Haoyun Fang, David Greening, Dilek Colak

**Affiliations:** 1 Center for Neurogenetics, Feil Family Brain and Mind Research Institute, Weill Cornell Medical College, Cornell University, New York City, NY USA; 2 Metabolomics Laboratory, Department of Biochemistry and Molecular Pharmacology, New York University, New York City, NY USA; 3 Molecular Proteomics Group, Baker Institute, Melbourne, VIC Australia; 4 La Trobe Institute for Molecular Sciences, La Trobe University, Melbourne, VIC Australia; 5 Gale and Ira Drukier Institute for Children’s Health, Weill Cornell Medical College, Cornell University, New York City, NY USA

**Keywords:** Schizophrenia, Neuroscience

## Abstract

It is widely accepted that narcotic use during pregnancy and specific environmental factors (e.g., maternal immune activation and chronic stress) may increase risk of neuropsychiatric illness in offspring. However, little progress has been made in defining human-specific in utero neurodevelopmental pathology due to ethical and technical challenges associated with accessing human prenatal brain tissue. Here we utilized human induced pluripotent stem cells (hiPSCs) to generate reproducible organoids that recapitulate dorsal forebrain development including early corticogenesis. We systemically exposed organoid samples to chemically defined “enviromimetic” compounds to examine the developmental effects of various narcotic and neuropsychiatric-related risk factors within tissue of human origin. In tandem experiments conducted in parallel, we modeled exposure to opiates (μ-opioid agonist endomorphin), cannabinoids (WIN 55,212-2), alcohol (ethanol), smoking (nicotine), chronic stress (human cortisol), and maternal immune activation (human Interleukin-17a; IL17a). Human-derived dorsal forebrain organoids were consequently analyzed via an array of unbiased and high-throughput analytical approaches, including state-of-the-art TMT-16plex liquid chromatography/mass-spectrometry (LC/MS) proteomics, hybrid MS metabolomics, and flow cytometry panels to determine cell-cycle dynamics and rates of cell death. This pipeline subsequently revealed both common and unique proteome, reactome, and metabolome alterations as a consequence of enviromimetic modeling of narcotic use and neuropsychiatric-related risk factors in tissue of human origin. However, of our 6 treatment groups, human-derived organoids treated with the cannabinoid agonist WIN 55,212-2 exhibited the least convergence of all groups. Single-cell analysis revealed that WIN 55,212-2 increased DNA fragmentation, an indicator of apoptosis, in human-derived dorsal forebrain organoids. We subsequently confirmed induction of DNA damage and apoptosis by WIN 55,212-2 within 3D human-derived dorsal forebrain organoids. Lastly, in a BrdU pulse-chase neocortical neurogenesis paradigm, we identified that WIN 55,212-2 was the only enviromimetic treatment to disrupt newborn neuron numbers within human-derived dorsal forebrain organoids. Cumulatively this study serves as both a resource and foundation from which human 3D biologics can be used to resolve the non-genomic effects of neuropsychiatric risk factors under controlled laboratory conditions. While synthetic cannabinoids can differ from naturally occurring compounds in their effects, our data nonetheless suggests that exposure to WIN 55,212-2 elicits neurotoxicity within human-derived developing forebrain tissue. These human-derived data therefore support the long-standing belief that maternal use of cannabinoids may require caution so to avoid any potential neurodevelopmental effects upon developing offspring in utero.

## Introduction

Cortical development is regulated by numerous mechanisms that discretely ensure that a series of temporally ordered events unfold in the correct order, generate the correct cell-types, and ultimately generate morphologically patterned tissue. This requires intact programming that emerges from a wide berth of biological processes, including the expression of specific genes [1] and molecules [2], patterns of sustained metabolic activity [3], the prevention of DNA damage [4], ongoing cell-cycle dynamics [5], regulation of cell survival mechanisms [6, 7], as well as orchestrated cell fate decision making [8]. Should any of these processes become altered during in utero cortical development, neocortical neurogenesis may become attenuated and this may yield developmental disorders, disruptions and/or delays. Thus, in utero brain development remains a critical period of risk for numerous neurodevelopmental disorders [9], including autism [10–12], and schizophrenia [13–16].

While risk for highly penetrant cases of neuropsychiatric illness are considered to predominantly arise from latent genetic risk, epidemiological evidence indicates that environmental factors also contribute risk to neurodevelopmental aberrations that may be linked with disease [9, 11–14]. This includes in utero narcotic and/or substance use, maternal immune activation [9, 13–15], as well as other risk factors such as chronic stress [11, 16]. The effects on the brain of most commonly abused drugs of abuse (e.g., cannabinoids or opiods) and bioactive substances (e.g., nicotine in cigarettes [17]) found in consumer products have typically been studied in adolescent and/or adult systems, models, or participants. However, many of these findings are likely to remain relevant to fetal neurodevelopment. For example, nicotine binds cognate nicotinic acetylcholine receptors which are known to affect neural activity (e.g., spike-timing dependent plasticity [18, 19]) and neuronal survival in rodents [20]. In addition, prenatal nicotine exposure has been associated with spine and other, broader, neuroanatomical changes in rats [21]. Similarly, ethanol (as a proxy for alcohol exposure) modulates cortical neuronal excitability [22–24], progenitor cell proliferation [25–27], cortical neuron migration [28], spine density [29, 30], and can cause Fetal Alcohol Syndrome (FAS) [31]. Similarly, opioids have been suggested to alter prenatal neural proliferation [32], induce alterations in growth factor expression (e.g., BDNF [33]), as well as modulate neurogenesis [33, 34]. Notably, cognate receptors for opiates (for e.g., both µ‐ and κ‐opioid receptors) are expressed by neural stem cells and progenitors [35–38]. Due to increasing opioid abuse within communities, there have been both concomitant increases in methadone treatment of opiate dependence during pregnancy as well as rising cases of Neonatal Abstinence Syndrome (NAS) in offspring [38]. Marijuana has become the most commonly abused drug during pregnancy within the United States [39]. Since the endogenous endocannabinoid system is known to be important for both prenatal and postnatal brain development [40], there is particular urgency in understanding how cannabinoids may regulate neurodevelopment. However, due to both increasing recreational use and legalization efforts, it is important to generate appropriate datasets that provide evidence-based guidance regarding public health practice so not to unnecessarily stigmatize recreational marijuana use where legal. The primary psychoactive and molecular constituent of marijuana is Δ^9^-tetrahydrocannabinol, which functions via activation of cannabinoid type 1 (CB_1_) receptors [41]. Indeed, CB_1_ receptors are found during early phases of brain development [42, 43], and are functional as shown by their activation in response to treatment with the cannabinoid CB_1_ agonist and mimetic WIN 55,212-2 [44]. Consequently cannabinoid receptors have been implicated in numerous biological pathways essential for fetal brain development such as proliferation, migration, and neuronal synaptogenesis (for comprehensive reviews, see [42, 43]). Likewise, synthetic cannabinoid exposure is also a rising issue amongst pregnant women and may also result in prenatal brain alterations [45]. Therefore, understanding the effects of prenatal drug use in a systematic format specifically within human tissue remains an issue of fundamental public health importance.

Beyond substance use/abuse during pregnancy, there is also a rich literature which indicates that environmental risk factors may modulate neurodevelopment and increase risk of specific disorders [9] including autism [10–12] and schizophrenia [13–16]. The Maternal Immune Activation (MIA) model has consequently become a leading hypothesis for autism and schizophrenia that transects brain development, neurodevelopmental disorders, and psychoneuroimmunology [46, 47]. The mechanisms responsible for neurodevelopmental alterations induced by MIA are diverse and likely involves numerous mechanistic intermediaries [48] that may be both time-specific (i.e., dependent upon the neurodevelopmental timing of insult [49–52]) as well as maturation-dependent (i.e., an age-dependent emergence of a phenotype [53]). Recent work has shown that Interleukin 17a (IL17a) principally mediates the neurodevelopmental effects of MIA on the developing cortex [54]. Specifically, IL17a mediates alterations in cortical neuron numbers and organization as well as autism-related behavior in offspring, which could be prevented via attenuation of IL17a in vivo [54]. Another environment-related risk factor for the developing brain is prenatal stress exposure, which is both common and has been associated with various fetal neurodevelopmental and birth outcomes [54]. Pivotal to this is the ability of stress/trauma to potentially modify the expression of neurodevelopment-related genes [55] including growth factors essential for brain assembly such as BDNF [56]. Indeed, in utero exposure to dexamethasone has been shown to disrupt the radial migration of neurons within the developing cortex [57]. Antenatal glucocorticoid therapy (e.g., with betamethasone) also results in lower whole cortex convolution and smaller brain surface area relative to age-matched infant controls [58]. Glucocorticoid receptors have also been shown to potentially underlie critical periods of stress vulnerability during postnatal cortical development and maturation [56, 59, 60]. Indeed, prenatal stress experiences may modify behavior [61] as well as elevate risk of autism [11], depression [62, 63], anxiety [61, 64], and schizophrenia [16, 65]. However, the signatures associated with in utero stress exposure within human tissue still remain largely unknown due to a longstanding inability to ethically access and manipulate *developing* human neural tissue.

Human-derived Induced Pluripotent Stem Cell (iPSC) technology now offers the potential to study human neurodevelopmental phenotypes via the generation of self-developing and self-organizing neural tissue [66]. These three-dimensional (3D) cultures, known as organoids, mimic the developing cerebral cortex and are approximate models of trimester one of pregnancy [66]. Importantly, a variety of studies have shown that various 3D organoid models faithfully recapitulate aspects of transcriptional [67], epigenetic [68, 69], and proteome programing [70] of fetal brain development. They also exhibit morphology (e.g., ventricles and ventricular zones, as well as developing cortical plates) [66] and cell-types/cellular diversity that is consistent with early corticogenesis [71–73]. Organoids are also typically enriched for various neuronal progenitor and early-born neuron populations that are consistent with early cortical development [71–73]. Organoids therefore provide a promising platform to ethically study developing neural tissue of human origin under controlled laboratory conditions [74]. As such, 3D human-derived models of the developing brain have become a viable model [75] from which to devolve human-specific mechanisms of brain development [76], evolution [77], and diseases [78] such as autism [79–81] and schizophrenia [70, 82–84]. Therefore, human-derived organoids now provide a platform and method from which to experimentally disentangle the effects of neuropsychiatric risk factors, including drug use, in developing neural tissue of human origin.

Here, we sought to determine the prenatal effects of various drug and neuropsychiatric risk factors on early corticogenesis within human-derived tissue. To do this, we generated 3D dorsal forebrain (cortical) organoids (see Methods and [71]) from human iPSCs, and systematically treated samples with various enviromimetic agents. In tandem experiments conducted in parallel, we modeled exposure to opiates (μ-opioid agonist endomorphin), cannabinoids (WIN 55,212-2), alcohol (ethanol), smoking (nicotine), chronic stress (human cortisol), and maternal immune activation (human IL17a) by chronically treating human-derived organoids for 7 Days In Vitro (DIV) before conducting a range of high-throughput analytical assays. This included 16-Plex Tandem Mass Tag (TMT) Liquid-Chromatography/Mass-Spectrometry (LC/MS) proteomics, state-of-the-art hybrid MS metabolomics, single-cell DNA content analysis for cell-cycle determination, a multi-panel flow cytometry assay for cell death and DNA damage, and a pulse-chase neocortical neurogenesis assay. These analyses revealed both convergent and divergent signatures within human-derived dorsal forebrain organoids between enviromimetic treatment groups, and unbiasedly identified that the cannabinoid agonist WIN 55,212-2 is a particularly noxious agent during cortical development.

## Results

### Generating 3D forebrain organoids for enviromimetic modeling of narcotic and neuropsychiatry-related risk factors

**Fig. 1:**
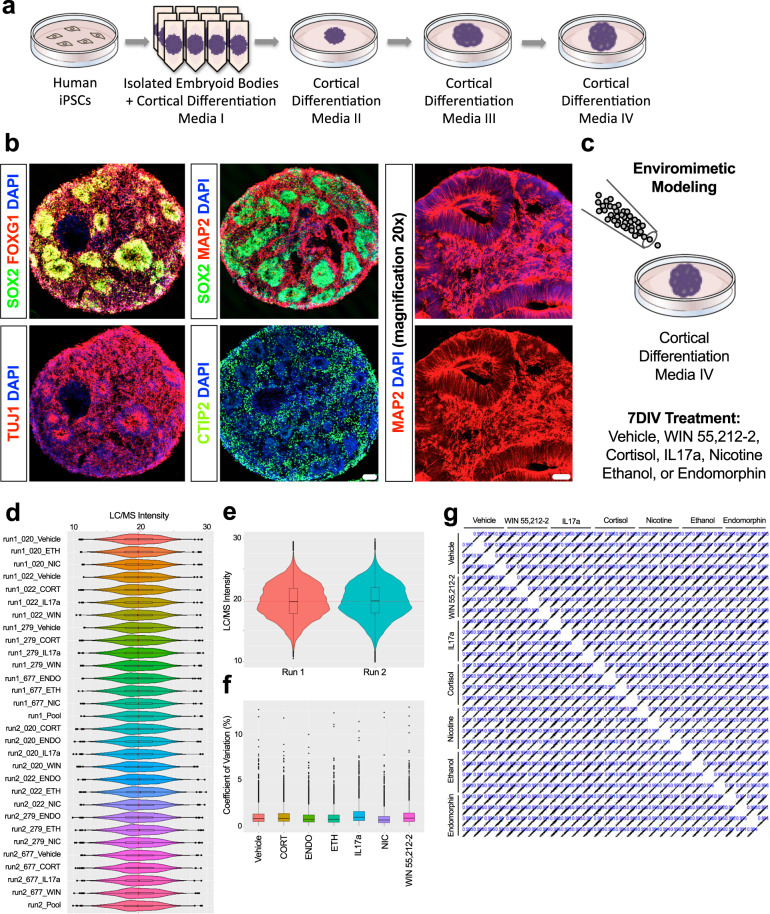
Enviromimetic forebrain organoids exhibit robust reproducibility. **a** Schematic of Human-Derived Dorsal Forebrain Organoid Culturing Pipeline. To generate dorsal forebrain organoids, iPSCs from healthy adult donors were expanded, dissociated into a single-cell suspension, and transferred to ultra-low adherence V-bottom plates. This allowed dissociated iPSCs to proliferate in-suspension to yield highly uniformed (i.e., size constricted) embryoid bodies that were morphologically consistent across wells, plates, and biological donors. In subsequent culturing stages, embryoid bodies were transferred to ultra-low adhesion 6 cm dishes and sequentially transferred through a series of cortical differentiation media (CDM2–4). **b** Human-Derived Organoids Exhibit Robust Neural Induction and Prototypical Forebrain Developmental Markers Consistent with Early Corticogenesis. As a quality control checkpoint, a pseudorandom assortment of human-derived dorsal forebrain organoids were sampled, drop-fixed, cryosectioned, and immunostained for prototypical forebrain developmental markers. Given the pre-established construct validity of this model, we focused our quality control assessments on the expression of neural stem cells (SOX2 + ; green), forebrain-specific progenitors (FOXG1 + ; red), pan-neuronal markers (TUJ1 + and MAP2 + ; both red in relevant panels), and forebrain-specific early-born neurons (CTIP2 + ; green). This evaluation revealed the expected morphologies of forebrain-specific organoids (e.g., the presence of ventricles, ventricular zones, and developing cortical plates). In addition, all neural-related and forebrain-specific antigens were observed in cultures from all biological donors, indicating the successful assumption of a restricted dorsal forebrain fate. **c** Schematic of Enviromimetic Treatment Regime. To model exposure to our various narcotic and neuropsychiatric-related enviromimetic risk factors, we chronically treated human-derived dorsal forebrain organoids with a variety of widely-utilized compounds used to study the acute and/or developmental effects of drug exposure and/or environmental risk factors related with mental illnesses. More specifically, this comprised a 7DIV exposure to the cannabinoid receptor agonist WIN 55,212-2, the maternal risk factor IL17a, the human stress hormone cortisol, nicotine, ethanol, and the μ-opioid agonist endomorphin. To ensure consistency across conditions and biological donors, treatments were simultaneously administered to parallel batches of organoids that had been generated from the same originating pool of iPSCs for each human donor. Likewise, all human donor samples were cultured simultaneously and maintained in parallel. **d** Box plots of Raw TMT-LC/MS Intensities Indicates Robust Reproducibility of 3D Organoid Cultures Across Biological Donors and Replicates. To unbiasedly establish the reproducibility of our culturing pipeline, we adapted cutting-edge Tandem Mass Tag (TMT) chemistry and high-performance liquid-chromatography/mass-spectrometry (LC/MS) to computationally map the molecular composition of human-derived 3D dorsal forebrain organoids from an isobarically-barcoded condensed pool. This real-time detection strategy thus reduces technical noise and variation, and enables rapid high-content sampling of our various treatment conditions. Our TMT-LC/MS dataset identified 40,452 peptides that could be mapped to 5,120 proteins. Based on statistical thresholds and expression levels, 4857 of these proteins could be quantified. Here we present raw TMT-LC/MS intensities for all individual human donors including all possible permutations relating to our enviromimetic treatment conditions. As shown, the distribution of TMT-LC/MS intensities was extremely similar across all groups and biological donors, indicating robust reproducibility of our 3D dorsal forebrain organoids across all biologics and conditions. **e** Violin Plots of Raw TMT-LC/MS Intensities Reveal No Evidence of Batch Variance. Because of the large number of samples studied here (*n* = 30 samples in total including pools/internal references, comprising *n* = 28 human samples for analysis), our TMT-LC/MS analysis had to be split into two 16-plex runs. We therefore sought to determine the reproducibility of our raw TMT-LC/MS datasets across distinct batches of organoids, donors, and treatment conditions. Splitting raw TMT-LC/MS intensities as a function of batch revealed that raw TMT-LC/MS intensities exhibited remarkably similar distributions as denoted in violin plots provided in (**1e**). **f** Comparable Coefficients of Variation Across Protein Intensities of All Groups. We next sought to compute coefficients of variation for protein intensities to examine the relative variability within and between our various narcotic and neuropsychiatric-related enviromimetic treatment groups. This analysis once more revealed that all groups exhibited similar coefficients of variation in protein expression, further indicating that our 3D dorsal forebrain cultures remained reproducible even after accounting for divergences in treatment allocation. **g** Correlation Matrix of All Donors and Groups Confirm Sample Reproducibility. Last, generation of a stringent correlation matrix revealed that there was robust correlation within treatment groups (oftentimes, *r*^2^ > 0.99) as expected. In sum, this unbiased statistical analysis confirmed that our culturing pipeline yielded reproducible 3D dorsal forebrain tissue across donors, conditions, and independent batches. For all panels, each donor sample was treated with all experimental compounds. This yielded *n* = 4 iPSC donors x *n* = 7 treatment groups for a total *n* of = 28 experimental samples for LC/MS analysis. Scale bar in *b* = 100 μm for whole organoid images and 60 μm for ×20 magnification of MAP2 immunostaining.

To model human cortical development, we generated self-assembling, self-organizing, and self-maturing 3D organoids that are designed to acquire a dorsal forebrain (i.e., cortical) fate. To do this, we adapted an expedited version [70] of a nascent organoid protocol [71] that reportedly exhibits greater reproducibility than preceding models (e.g., [66]). In rolling pseudorandom quality control assessments of organoids, samples from all donors exhibited evidence of early corticogenesis. This included morphological features such as ventricles, ventricular zones, and evidence of developing cortical fields. Immunostainings consequently confirmed robust neural induction (expression of SOX2 + neural stem cells as well as MAP2 + and TUJ1 + neurons) as well as the acquisition of a forebrain-specific fate (expression of forebrain-specific FOXG1 + progenitors and CTIP2 + early-born cortical neurons). Prototypical morphological features, expected cell-types, and the induction of forebrain-specific cortical transcription factors are shown via whole-organoid Immunostainings in Fig. 1b.

### Enviromimetic modeling of narcotic and neuropsychiatric-related risk factors

To model exposure to our various narcotic and neuropsychiatric-related risk factors, we chronically treated human-derived dorsal forebrain organoids with an array of enviromimetic treatments commonly utilized within preclinical neuropsychiatry research. More specifically, after culturing organoids for 30–35 DIV, human-derived dorsal forebrain organoids were exposed to one of a variety of enviromimetic treatments that was added to CDM4 media for an additional 7 DIV (see schematic in Fig. 1c). This comprised exposure to the cannabinoid receptor agonist WIN 55,212-2, the maternal risk factor IL17a, the human stress hormone cortisol, nicotine, ethanol, or the μ-opioid agonist endomorphin (see “Methods” for more information). After this 7 DIV exposure period, organoids were processed and prepared for one of several streams of analysis including liquid-chromatography/mass spectrometry (LC/MS) proteomic profiling, hybrid MS-based metabolomic profiling, high-throughput flow cytometry for unbiased assessments of apoptosis and DNA-damage, single-cell DNA content analysis for cell-cycle and DNA fragmentation analysis, and/or immunohistochemistry for neuronal quantifications. This approach therefore allowed us to ethically examine the neurodevelopmental effects of narcotic and neuropsychiatric-related risk factors in 3D human-derived forebrain tissue under controlled laboratory conditions.

### Establishing the reproducibility of 3D human-derived dorsal forebrain organoids

Before conducting experiments, we first sought to ensure that our human-derived dorsal forebrain organoids exhibited sufficient reproducibility across all treatment conditions and donors. In two separate TMT 16-plex pools, a total of 30 samples (comprising *n* = 28 experimental samples and *n* = 2 internal references/pools for data normalization) were barcoded with TMT reagents, condensed into a single sample, and subjected to simultaneous detection via LC/MS. In total, this approach identified 40,452 peptides with average sequence coverage of 20.8%. These peptides were subsequently mapped to 5120 proteins of which 4857 could be quantified (94.86% of proteins identified were quantifiable). Of these proteins, 3711 were common to all donor and treatment samples and required no further imputation for statistical analysis.

To ensure that our forebrain enviromimetic organoid model exhibited sufficient reproducibility across treatment conditions, we first conducted a statistical analysis of sample variation. Visual inspection of all raw data points per individual sample revealed that all samples exhibited similar LC/MS intensity distributions for detected proteins (Fig. 1d). To evaluate batch effects data were also split by each TMT-LC/MS run and graphed as violin plots. No evidence of technical batch variation was detected when examining raw TMT-LC/MS intensities for all 28 experimental samples (Fig. 1e). An analysis of treatment-group variation also revealed no evidence of large deviations in coefficients of variation when all data points were examined and mapped as boxplots split by conditions (Fig. 1f). Coefficients of proteome variation revealed that sample variance was less than 1% of the median within treatment groups, and exhibited a <10% range. In addition, generation of a stringent correlation matrix revealed that there was robust similarity within treatment groups (*r*^2^ > 0.99) as expected (Fig. 1g). In sum, this statistical analysis confirmed that our culturing pipeline yielded reproducible 3D dorsal forebrain tissue across donors, conditions, and independent batches.

### Developmental alterations within the proteome of human-derived organoids following treatment with narcotic and neuropsychiatry-related enviromimetics

We next sought to statistically identify novel molecular alterations between and within our 6 different narcotic and neuropsychiatric risk factor treatment groups. To do this, we first clustered our samples based on a structural equation modeling approach that yielded a principal components solution based on the expression of 3,711 proteins. To unbiasedly identify an effect of treatment group on protein expression, we utilized this dataset for all further downstream analyses for novel factor identification.

**Fig. 2:**
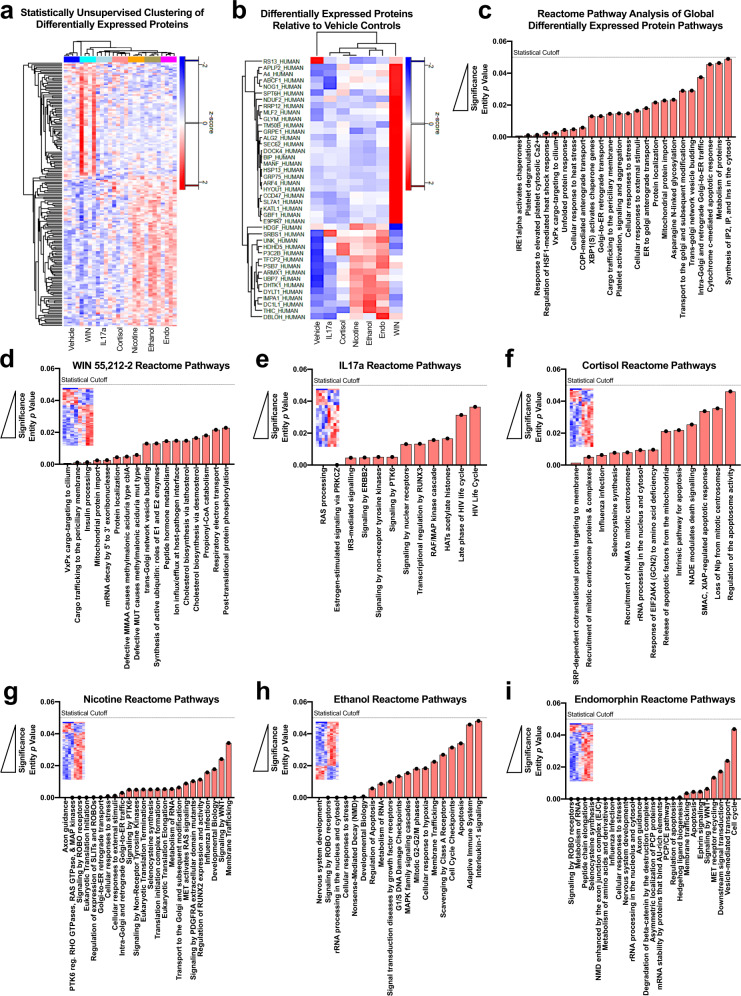
Proteome and reactome pathway alterations in human-derived forebrain organoids across narcotic and neuropsychiatric-risk enviromimetic treatment groups. **a**–**c** Statistically unsupervised analysis of TMT-LC/MS proteomics comprising all samples. All 28 of our TMT-LC/MS samples were first subjected to a statistically unbiased analysis that did not consider a priori hypotheses or the quasi-independent nature of each of our treatment conditions. This yielded a clustering solution (**a**) that identified differential expression of 41 specific proteomic targets that differed from vehicle controls (**b**). These proteins were correspondingly mapped to their Reactome pathways of origin (**c**). Note, the ordering of groups for presentation purposes are assigned based on the results of statistically unsupervised clustering. **d**–**i** Reactome pathway analysis split by narcotic and neuropsychiatric-related treatment. Analysis of the Reactome pathways split by our individual treatments revealed novel enrichment patterns for each narcotic compound and enviromimetic treatment conditions. As described in-text, WIN 55,212-2, IL17a, and cortisol exhibited the most distinct Reactome pathway patterning, indicating a greater degree of divergence in pathway alterations. Contrary to this, our nicotine, ethanol, and endomorphin treatment groups tended to exhibit a closer degree of convergence. Specifically, these groups tended to exhibit greater enrichment for neurodevelopment-related Reactome pathways including factors mapped to “nervous system development”, “ROBO receptor signaling”, and/or “axon guidance”. However, each of these groups still maintained unique patterns of Reactome pathway enrichment, which perhaps indicates a common role in their ability to alter neurodevelopmental factors albeit via divergent intermediaries. For all panels, each donor sample was treated with all experimental compounds (4 iPSC donors x 7 groups = 28 total samples for analysis).

A global analysis of all TMT-LC/MS intensities, which did not stratify individual group for a priori hypothesized effects, revealed that 175 proteins differed in at least one group within our total dataset (Fig. 2a; see also Supplementary Table 1). Of these 175 proteins, a stratified analysis revealed that only 41 proteins were specifically different in our 6 enviromimetic treatment groups relative to our vehicle-treated control group (Fig. 2b). In this total pool, WIN 55,212-2 exhibited the most distinct expression profile and did not cluster further with the other narcotic treatment groups. Contrary to this, our neuropsychiatry enviromimetics (both IL17a and cortisol) exhibited similar overall proteome differences and clustered together, whereas nicotine, ethanol, and endomorphin were more similar than any other combination of groups (Fig. 2a-b). Further examination of the 41 differentially expressed proteins identified in the total data pool yielded several notable observations. This list revealed enrichment for factors involved in hypoxia (e.g., hypoxia up-regulated protein 1, or HYOU1), cellular stress responses (e.g., the heatshock proteins HSPA13 and HSPA9), and amyloid-related proteins. Notably, this included amyloid-like protein 2 (APLP2) and the amyloid beta precursor protein (APP). There was also enrichment for a novel growth factor (i.e., mesencephalic astrocyte-derived neurotrophic, or MANF) and proteins localized to, or involved in the functioning of, mitochondria and/or regulation of other cellular metabolic functions (see Table 1). A complete list of these 41 proteins identified in our global/unbiased analysis are listed within Table 1 and, for brevity, a summary of common targets is provided for each individual group in Table 2. Notably, the cannabinoid agonist WIN 55,212-2 and the μ-opioid agonist endomorphin tended to exhibit a more similar overlap in proteome targets, whereas our nicotine, ethanol and cortisol treatment groups tended to be more similar in their combination of proteins targeted (see Table 2).

**Table 1:** Top differentially expressed proteins (relative to Ctrls) detected via TMT-LC/MS.

Short protein name	Long protein name	Uniprot accession	ANOVA *p* value
NDUFAF2	NADH Dehydrogenase (Ubiquinone) 1 Alpha Subcomplex Assembly Factor 2	Q8N183	0.000481025
TMEM50B	Transmembrane Protein 50B	P56557	0.000548505
IMPA1	Inositol Monophosphatase 1	P29218	0.001902383
ARMCX1	Armadillo Repeat-Containing X-Linked Protein 1	Q9P291	0.002196252
GBF1	Golgi-Specific Brefeldin A-Resistance Guanine Nucleotide Exchange Factor 1	Q92538-3	0.002520107
HDHD5	Haloacid Dehalogenase-Like Hydrolase Domain-Containing 5	Q9BXW7-2	0.00307041
GTPBP4	Nucleolar GTP-Binding Protein 1	Q9BZE4	0.00324609
ARF4	ADP-Ribosylation Factor 4	P18085	0.003766362
GRPEL1	GrpE Protein Homolog 1, Mitochondrial	Q9HAV7	0.004413199
ACAT2	Acetyl-CoA Acetyltransferase, Cytosolic	Q9BWD1	0.004730022
MANF	Mesencephalic Astrocyte-Derived Neurotrophic Factor	P55145	0.005179981
DOCK4	Dedicator of Cytokinesis Protein 4	Q8N1I0–2	0.00538423
TXNRD1	Thioredoxin-Disulfide Reductase	E9PIR7	0.005572821
DIABLO	Diablo Homolog, Mitochondrial	Q9NR28-2	0.00627476
HSPA13	Heat Shock 70 kDa Protein 13	P48723	0.00627476
TFCP2	Alpha-Globin Transcription Factor CP2	Q12800-2	0.00639284
DYNLT1	Dynein Light Chain Tctex-Type 1	P63172	0.00671083
HDGF	Hepatoma-Derived Growth Factor	P51858	0.007032222
PSMB7	Proteasome Subunit Beta Type-7	Q99436	0.007289525
UNK	RING Finger Protein Unkempt Homolog	Q9C0B0	0.008045913
HYOU1	Hypoxia Up-Regulated Protein 1	Q9Y4L1	0.008687815
SEC62	Translocation Protein SEC62	Q99442	0.010325685
APLP2	Amyloid-Like Protein 2	Q06481-4	0.013408141
USP7	Ubiquitin Carboxyl-Terminal Hydrolase 7	Q93009-3	0.015504379
KATNAL1	Katanin p60 ATPase-Containing Subunit A-Like 1	Q9BW62	0.015622147
HSPA5	Endoplasmic Reticulum Chaperone BiP	P11021	0.015742232
DYNC1LI1	Cytoplasmic Dynein 1 Light Intermediate Chain 1	Q9Y6G9	0.016475164
ABCF1	ATP-Binding Cassette Sub-Family F Member 1	Q8NE71-2	0.016611257
SHMT2	Serine Hydroxymethyltransferase, Mitochondrial	P34897-3	0.018482781
DHTKD1	Probable 2-Oxoglutarate Dehydrogenase E1 Component DHKTD1, Mitochonondrial	Q96HY7	0.018748869
SLC7A1	High Affinity Cationic Amino Acid Transporter 1	P30825	0.021172737
PIK3C2B	Phosphatidylinositol 4-Phosphate 3-Kinase C2 Domain-Containing Subunit Beta	O00750	0.021899148
SORBS1	Sorbin and SH3 Domain-Containing Protein 1	Q9BX66-4	0.024998617
HSPA9	Stress-70 Protein, Mitochondrial	P38646	0.027136132
CCDC47	Coiled-Coil domain-Containing Protein 47	Q96A33	0.028644274
ALG2	Alpha-1,3/1,6-Mannosyltransferase ALG2	Q9H553	0.030668953
APP	Amyloid-Beta Precursor Protein	P05067-11	0.031024857
SUPT6H	Transcription Elongation Factor SPT6	Q7KZ85	0.033263913
RRP12	RRP12-Like Protein	Q5JTH9-2	0.033924326
MLF2	Myeloid Leukemia Factor 2	Q15773	0.043882525
RPS13	40 s Ribosomal Protein S13	P62277	0.047438594

**Table 2:** Common proteome alterations detected via global analysis of TMT-LC/MS data.

		Enviromimetic drug/risk factor treatment group					
Protein name	Uniprot accession	WIN	IL17a	CORT	Nicotine	Ethanol	Endomorphin
ADD1	P35611	↓					**↑**
POLR3C	Q9BUI4	**↑**					**↑**
TMEM50B	P56557	**↑**					↓
NELFCD	Q8IXH7	**↑**					**↑**
KPNA6	O60684	**↑**					**↑**
MTX2	Q75431	**↑**					**↑**
CLPTM1	Q96005	**↑**					**↑**
APP	P05067	**↑**					**↑**
FXR2	P51116	**↑**			**↑**	**↑**	**↑**
TFCP2	Q12800	**↑**	**↑**		**↑**	**↑**	**↑**
SUPT6H	Q7KZ85	**↑**					**↑**
APLP2	Q06481	**↑**		**↑**			**↑**
USP7	Q93009	**↑**			**↑**	**↑**	**↑**
TIMM21	Q9BVV7	**↑**					**↑**
APMAP	Q9HDC9	**↑**			**↑**	**↑**	**↑**
SRP19	P09132	**↑**		↓			
RPS27L	Q71UM5	↓		↓			
LSM3	P62310	**↑**		**↑**	**↑**		**↑**
TNPO3	Q9Y5L0	**↑**		**↑**		**↑**	**↑**
SAP30BP	Q9UHR5	**↑**		**↑**	**↑**		
APLP2	Q06481	**↑**		**↑**			**↑**
TMEM209	Q96SK2	**↑**		**↑**	**↑**		
RPL36	Q9Y3U8			↓	↓	↓	↓
RPS13	P62277			↓	↓	↓	↓
CDC42BPB	Q9Y5S2			**↑**	**↑**	**↑**	**↑**
FMNL2	Q96PY5			**↑**	**↑**	**↑**	**↑**
HDHD5	Q9BXW7			**↑**	**↑**	**↑**	**↑**
ZFYVE1	Q9HBF4		**↑**	**↑**	**↑**	**↑**	
ARMCX1	Q9P291			**↑**	**↑**	**↑**	**↑**
DHTKD1	Q96HY7			**↑**	**↑**	**↑**	**↑**

Because each of our treatment groups represent quasi-independent studies, the proteins identified in our global/unbiased analysis of the dorsal forebrain organoid proteome did not comprise specific pairwise comparisons between individual groups and vehicle-treated controls. This is of note, as this was the primary aim of the current study. To address this, we conducted a further analysis of our TMT-LC/MS intensities stratified specifically by each of our individual treatment groups. In fact, this further analysis of group-by-group differences led us to identify a greater number of differentially expressed proteins (see also Supplementary Table 2). Specifically, we identified 422 distinct proteins that significantly differed in their expression levels across various treatment conditions when each was independently compared against our vehicle-treated control organoids (see also Supplementary Table 3). Broadly, we found that our narcotic mimetic treatments tended to yield a broader spectrum of protein expression changes (endomorphin: *n* = 196 proteins, nicotine: *n* = 131 proteins, ethanol*: n* = 149 proteins, and WIN 55,212-2: *n* = 84 proteins, respectively). Contrary to this, our “*environmental*” neuropsychiatric risk factor treatments (cortisol: *n* = 49 proteins, and IL17a *n* = 19 proteins, respectively) tended to exhibit fewer overall protein alterations within human-derived dorsal forebrain organoids. A list of differentially expressed proteins in group-segregated pairwise analysis is provided in Table 3. Many of these proteins were differentially expressed across two or more treatment groups, and these common alterations are further summarized in Supplementary Table 4. However, each of our narcotic and neuropsychiatric-related treatments also resulted in unique, non-overlapping, proteome alterations (see Table 3 and Supplementary Table 4).

**Table 3:** Group-specific protein alterations via pairwise analysis of TMT-LC/MS data.

Enviromimetic drug/risk factor treatment group						
WIN 55,212-2	IL17A	Cortisol	Nicotine	Ethanol	Endomorphin	
**↑** TXNRD1	**↑** GPR89A	**↑** NDUFB9	**↑** ROBO1	**↑** ELOF1	**↑** MTHFD1L	**↑** EFNB3
**↑** UBA6	**↑** SUB1	**↑** PBDC1	**↑** FSD1L	**↑** MPP2	**↑** MAP4K4	**↑** NEDD8
**↑** AT2C1	**↑** DR1	**↑** RBM28	**↑** ELAVL2	**↑** PHF14	**↑** MYO18A	**↑** TBC1D9B
**↑** TRMT10A	**↑** GATD1	**↑** FLYWCH2	**↑** CAMK2G	**↑** NELFB	**↑** TSEN34	**↑** SLC27A4
**↑** TSPAN3	↓ NEBL	**↑** ROCK2	**↑** CNTN2	**↑** PAK1	**↑** GATAD2B	**↑** EDC4
**↑** YIPF4	↓ SUPT4H1	**↑** NME7	**↑** CHCHD3	**↑** PPP4R2	**↑** EIPR1	**↑** LARP4
**↑** TIMM17B	↓ DIS3	**↑** RIF1	**↑** NDRG3	**↑** NRCAM	**↑** PPIL3	**↑** TAOK1
**↑** CST3		**↑** SLC16A2	**↑** GGA3	**↑** CLASP2	**↑** AARSD1	**↑** BPHL
**↑** GSPT1		**↑** ABCB6	**↑** DCAKD	**↑** DCTN6	**↑** MRPL39	**↑** SBF2
**↑** CPE		**↑** ZNF579	**↑** PRKRA	**↑** CTNND2	**↑** ARVCF	**↑** ZC3H7A
**↑** ARF4		**↑** PDLIM3	**↑** GNAI3	**↑** CFL1	**↑** NEFM	**↑** PTGR2
**↑** MMUT		**↑** GCC2	**↑** RBM25	**↑** HS1BP3	**↑** THY1	**↑** UBA3
**↑** SHMT2		**↑** NDUFA2	**↑** EIF2B2	**↑** YLPM1	**↑** TSG101	**↑** FAM172A
**↑** NAA10		↓ ARMC9	**↑** MCAM	**↑** MACF1	**↑** PRKAG1	**↑** UBTD2
**↑** TUFM		↓ INTS9	**↑** NOVA1	**↑** MPRIP	**↑** LRRC57	**↑** RTF1
**↑** SLC25A3		↓ VIRMA	**↑** EIF5	**↑** DOHH	**↑** EPS15L1	**↑** CLCC1
**↑** CPSF1		↓ NUP210	**↑** CDK16	**↑** DAZAP1	**↑** AGRN	**↑** TRIM2
**↑** TRIP12			**↑** INA	**↑** DPYSL4	**↑** PSMD14	**↑** EGLN1
**↑** SART3			**↑** CIAPIN1	**↑** SPTBN2	**↑** SDCBP	**↑** FAM234A
**↑** WDR43			**↑** DHX57	**↑** KIF5C	**↑** TRAFD1	**↑** WDR13
**↑** DHCR24			**↑** UBE2R2	**↑** RTN3	**↑** UQCRQ	**↑** DPH5
**↑** PCID2			**↑** TRMT10C	**↑** CTPS1	**↑** PLXNB2	**↑** MYG1
**↑** METTL3			**↑** NIT1	**↑** CTNNA2	**↑** ZW10	**↑** HMG20A
**↑** NDUFAF2			**↑** TTC5	**↑** ABCD3	**↑** NIPSNAP2	**↑** PHPT1
**↑** RDH11			**↑** TNPO1	**↑** CARS1	**↑** PDCD6	**↑** SH3BP4
**↑** GBF1			**↑** RSF1	**↑** PEX3	**↑** GCAT	**↑** UQCR10
**↑** EXOC4			**↑** GAN	**↑** TBL3	**↑** ATRN	**↑** VPS28
**↑** GTPBP4			**↑** ACTR10	**↑** DBN1	**↑** TTC4	**↑** VPS4A
**↑** NIF3L1			**↑** SACS	**↑** TBCEL	**↑** GNAI2	**↑** PACSIN2
**↑** GHITM			**↑** COPG2	**↑** CADM2	**↑** EIF2S1	**↑** PSMD13
**↑** EXD2			**↑** CNOT11	**↑** AMER2	**↑** NEFL	**↑** RABGAP1
**↑** BPNT2			**↑** MAGED1	**↑** GDAP1	**↑** GALT	↓ SELENOH
**↑** YARS2			↓ TTC28	**↑** DTD1	**↑** CNP	↓ EIF1AD
**↑** RRP15			↓ MTAP	**↑** CLIP2	**↑** G6PD	↓ RPL28
**↑** AUP1			↓ DOCK4	**↑** UBQLN2	**↑** FDPS	↓ DVL2
↓ DOCK1			↓ SYNCRIP	**↑** DCTN4	**↑** EIF2AK2	↓ RPL19
↓ ACSS3			↓ PDIA6	**↑** NOVA2	**↑** RAB6A	↓ RPL4
↓ MEX3A			↓ PDCD4	↓ RPS9	**↑** ATP2B4	↓ ARL1
↓ ENSA			↓ DIPK2A	↓ SENP3	**↑** OTX1	↓ RPL5
↓ FAM171A2				↓ NAA50	**↑** CRAT	↓ RPL34
↓ PDCD10				↓ RPL15	**↑** CRK	↓ NUP107
↓ ATOX1				↓ PLOD2	**↑** CRKL	↓ RPL23A
↓ SORBS2				↓ MEIS2	**↑** BRCC3	↓ PPP2CA
↓ STX8				↓ ATP1A1	**↑** DHPS	↓ HNRNPD
↓ TLK2				↓ S100A6	**↑** IST1	↓ NUFIP2
↓ MIF				↓ FBL	**↑** CACNA2D1	↓ BTF3L4
↓ PPT1				↓ FKBP2	**↑** RAB5B	↓ API5
↓ CNN3				↓ NMT1	**↑** COPS2	↓ LSM6
↓ HMGN3				↓ TSFM	**↑** NCALD	↓ LAS1L
↓ PKN2				↓ TPMT	**↑** SNRPF	
↓ LGALSL				↓ CASP7	**↑** RAC1	
↓ ZC3H13				↓ NUTF2	**↑** VAC14	
↓ MTDH				↓ RPL11	**↑** TRAF2	
↓ LSM2				↓ DDI2	**↑** IFIT5	
				↓ ISOC1	**↑** HNRNPD	
				↓ SFXN2	**↑** UBE4A	
					**↑** ELAVL3	
					**↑** LAGE3	
					**↑** CRYM	

In sum, only a modest number of proteins within the developing cortical proteome were found to be robustly responsive to our various narcotic and neuropsychiatric-related treatments. In addition, we discovered that there were various degrees of overlap between treatment conditions in human-derived dorsal forebrain organoids. This implicates that the differential expression of a common ensemble of proteome factors may partially underscore some of the molecular dysfunction induced by our enviromimetic treatments during early human brain development.

### Reactome modeling reveals biological pathways altered by enviromimetic treatments in human-derived organoids

To gain insight into the specific pathways and biological functions associated with our narcotic and neuropsychiatric-related treatments, we next applied Reactome pathway analysis to this list of candidate differentially expressed factors identified via TMT-LC/MS proteomics. This global analysis revealed that these proteins mapped to biological functions comprising IP^2^/IP and Ca^2+^ regulation, cellular responses to heat shock stress, cargo transport pathways including trafficking in and out of the Golgi apparatus, as well as pathways related to protein metabolism and mitochondrial-related (e.g., protein import) functionality (Fig. 2c). However, broadly speaking, a more targeted approach was necessary to statistically parse the individual contributions of specific treatment conditions.

As expected, parsing these differentially regulated proteins for alterations in Reactome pathways differences between our various narcotic and neuropsychiatric enviromimetic treatments revealed group-specific effects. A complete summary of differentially regulated biological processes and pathways between groups is visualized in Fig. 2d-i. Examination of top differentially enriched pathways revealed that human-derived dorsal forebrain organoids treated with the cannabinoid receptor agonist WIN 55,212-2 exhibited altered golgi apparatus VxPx cargo-targeting and trafficking to the cilium and periciliary membrane (FDR = 0.005 and 0.08, respectively; see Fig. 2d). The cilium functions as its own compartmentalized organelle, and is a region where membrane proteins become natively concentrated within the cell [85] that is also required for Sonic Hedgehog (Shh) signal transduction [86]. Not unexpectedly, cilium-related function and transport of membrane proteins has been implicated in both neurodevelopment and disease [86]. Contrary to this, the human maternal immune risk factor IL17a (Fig. 2e) was notably defined by enrichment for RAS processing (FDR = 0.002), which is important for regulation of the brain’s angiotensin system [87]. In addition, IL17a treatment resulted in enrichment for estrogen-stimulated signaling through Protein Kinase C (PKC) Zeta (FDR = 0.002; see Fig. 2e). PKCZ has a suggested role in regulating cell polarity during migration within developing neuroblasts [88] and anterior-posterior axon guidance derived from WNT and Pl3K signaling [89], indicating this pathway may influence normative neurodevelopmental processes within the developing forebrain. Chronic cortisol treatment (Fig. 2f) in human-derived dorsal forebrain organoids yielded enrichment for SRP-mediated protein translation within the endoplasmic reticulum as well as the recruitment of mitotic centrosome proteins and complexes. While the entity *p* values for these pathways remained low (*p* = 0.001 and 0.005, respectively) the FDR scores for these cortisol-related pathways were higher than for alterations observed in other groups (FDR = 0.133 for both pathways). Treatment of organoids with nicotine (Fig. 2g) yielded enrichment for numerous canonical pathways essential for normal cortical development, including axon guidance (FDR = 0.009), regulation of RHO/RAS Guanosine Triphosphatases (GTPases; FDR = 0.009), ROBO signaling (FDR = 0.009), and the initiation of eukaryotic protein translation (FDR = 0.01). Similarly, treatment of human-derived organoids with ethanol (Fig. 2h) also revealed enrichment for ROBO receptor signaling (FDR = 6.25E–05), which is important for cortical neurogenesis [90] and axonal guidance [91], as well as an array (~26 proteins) that have been broadly mapped as “nervous system development” factors (FDR = 3.61E–05). Ethanol also exhibited enrichment for cellular stress response factors (FDR = 0.002), Nonsense-Mediated mRNA Decay (NMD; FDR = 0.004), developmental factors (FDR = 0.01), and the regulation of apoptosis (FDR = 0.03). Last, treatment of human-derived dorsal forebrain organoids with the μ-opioid agonist endomorphin (Fig. 2i) also revealed enrichment for ROBO receptor signaling (FDR = 3.34E–05), RNA metabolism factors (FDR = 7.04E–05), exon-enhanced NMD of mRNA transcripts (FDR = 5.05E–04), cellular stress response factors (FDR = 7.02E–04), central nervous system development factors (FDR = 0.0027), and axon guidance factors (FDR = 0.003).

Similar to our analysis of individual proteomic targets, a comparison of Reactome pathway enrichment between our various treatment groups also revealed several noteworthy similarities. Namely, our ethanol, endomorphin, and WIN 55,212-2 treatment groups exhibited enrichment for various mRNA stability and degradation pathways (notably, NMD and 5ʹ to 3ʹ exoribonuclease mRNA degradation pathway factors). Similarly, treatment of human-derived dorsal forebrain organoids with ethanol, endomorphin, cortisol, and nicotine yielded enrichment for rRNA processing factors within the nucleolus and cytosol. Several groups also exhibited differential recruitment of pathways related to protein translation (e.g., nicotine, cortisol), as well as central nervous system development, ROBO receptor signaling, and/or axon guidance (e.g., ethanol, endomorphin, and nicotine). It was also not uncommon for groups to exhibit various degrees of enrichment for pathways involved in mitosis or cell cycle checkpoint activity (e.g., ethanol, endomorphin, and cortisol) as well as pathways involved in cellular stress, apoptosis, or hypoxia (e.g., ethanol, endomorphin cortisol, and nicotine). Of our 7 groups, the most distinct profile belonged to human-derived organoids treated with the cannabinoid agonist WIN 55,212-2, followed by organoids treated with human IL17a and cortisol. Contrary to this, our ethanol, endomorphin, and nicotine groups exhibited a greater number of similarities than any other combination of groups, indicating partially convergent molecular effectors and potentially similar pathways of action within 3D human-derived forebrain organoids.

### Metabolomic signatures of narcotic and neuropsychiatric enviromimetic treatments within human-derived forebrain organoids

Across our various proteomic datasets, many of our treatment groups exhibited enrichment for individual protein factors and/or Reactome pathways that have known roles in cellular metabolic and/or bioenergetic function. Therefore, to provide further unbiased systems level analysis of our enviromimetic treatments in human-derived dorsal forebrain organoids, we adapted a targeted hybrid metabolomics panel to map the developing organoid metabolome.

**Fig. 3:**
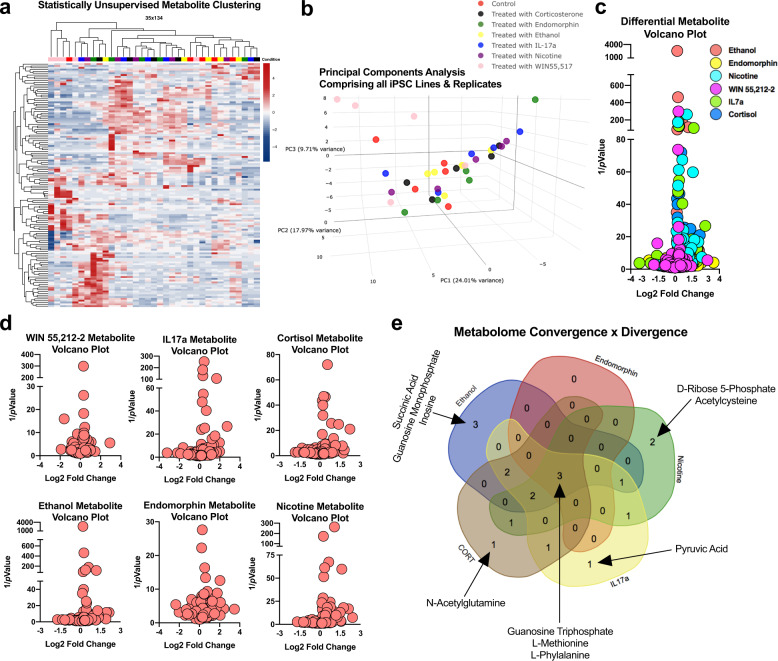
Hybrid metabolomics reveals novel metabolite alterations within enviromimetic forebrain organoids. **a**, **b** Statistically unsupervised analysis of treatment-induced metabolites. The metabolome of dorsal forebrain organoids exposed to our narcotic and neuropsychiatric-related treatments were first analyzed and in a statistically unsupervised format. This involved unbiased clustering of the metabolomic profiles of all samples (**a**). In addition, a statistically unsupervised principal components analysis was also conducted to visualize the distinctness of our various treatments within human-derived dorsal forebrain organoids (**b**). Note, the ordering of groups for presentation purposes are presented differ here due to the hierarchical relationships present between samples in these statistically-unsupervised clustering/principal components analyses. **c, d** Metabolomic Log2FC alterations visualized as split-axis volcano plots. Metabolic factors were next analyzed as a function of both their Log2FC and statistical significance. This yielded a global metabolite profile for all treatment conditions (see global volcano plot in (**c**)). Differential metabolite expression was subsequently split as a function of treatment group and was visualized as group-specific volcano plots that had been statistically compared against the metabolite profile of vehicle-treated controls (**d**). **e** Metabolome convergence and divergence across narcotic and neuropsychiatric-related risk factors in dorsal forebrain organoids. Further analysis of metabolomes revealed both common and unique metabolite alterations between human-derived dorsal forebrain organoids exposed to our different treatment conditions. This included differential expression of L-Phenylalanine in all treatment groups. The neurodevelopment-related metabolite Guanosine Triphosphate (GTP) was also differentially expressed in all treatment groups except those exposed to WIN 55,212-2. Because WIN 55,212-2 exhibited no further commonalities with our other groups, only the remaining 5 narcotic and neuropsychiatric-related treatment conditions were visualized (**e**). As discussed in-text, further analysis revealed that treatment of human-derived dorsal forebrain organoids with IL17a altered pyruvic acid expression, cortisol altered N-Acetylglutamine, nicotine altered D-Ribose 5-Phosphate and Acetylcysteine, and ethanol treatment yielded altered succinic acid, guanosine monophosphate, and inosine. Further discussion of metabolite data is provided in-text. Note that (**a**) and (**b**) share the same group legend, which is presented in (**b**). For all panels, each donor sample was treated with all experimental compounds. This yielded *n* = 5 iPSC donors x *n* = 7 treatment groups for a total *n* of = 35 experimental samples for LC/MS analysis.

First, imputed sample values were clustered via statistically unsupervised analysis to yield an unbiased visualization of drug and enviromimetic treatment effects (Fig. 3a). Clustering was subsequently cross-validated via another statistically unsupervised analysis that segregated samples into principal components within the broader dataset (Fig. 3b). Both of these unbiased analyses revealed that the metabolome was subtly altered relative to vehicle-treated controls. In subsequent group analyses, data were imputed against metabolite expression patterns in control samples, and analyzed for individual group differences based on Log2 fold change and *p* values (Fig. 3c–e). A summary of Log2Fold differences and *p* values, as well as similarities and differences in differentially expressed metabolites, are provided in Tables 4–5. Our analysis consequently led us to identify both the convergence of enviromimetic organoids upon alterations in certain metabolites, as well as unique divergences between groups. Typically speaking, there were 10–11 significantly altered metabolites in most groups. The exception was our endomorphin and WIN 55–212,2 treatment groups, which exhibited an alteration in just 3 factors which were also commonly altered in all other groups (i.e., these treatments did not exhibit any unique differentially expressed metabolites). This included significant alterations in the expression of amino acids (WIN 55–212,2: L-Tyrosine & L-Valine; Endomorphin: L-Methionine) and, in both groups, L-Phenylalanine.

**Table 4:** Differentially expressed metabolites in human enviromimetic organoids.

	Enviromimetic drug/Risk factor treatment group					
	WIN	IL17a	CORT	Nicotine	Ethanol	Endomorphin
3-Phosphoglyceric Acid		↓	↓			
Acetylcysteine				↓		
Allantoin		↓		↓		
Creatinine		↓	↓	↓	↓	
D-Ribose 5-Phosphate				↓		
Guanosine Monophosphate					↓	
Guanosine Triphosphate		↑	↑	↑	↑	↑
Inosine					↓	
L-Leucine		↓	↓		↓	
L-Methionine		↓	↓	↓	↓	↓
L-Phenylalanine	↓	↓	↓	↓	↓	↓
L-Tyrosine	↓	↓	↓	↓	↓	
L-Valine	↓	↓		↓	↓	
N-Acetylglutamine			↓			
N-Acetylornithine		↓	↓		↓	
Pyruvic Acid		↓				
S-Adenosylhomocysteine			↓	↓		
Succinic Acid					↓

**Table 5:** Statistics for metabolites altered across human-derived organoid groups.

	Enviromimetic Drug/Risk factor treatment group					
	WIN	IL17a	CORT	Nicotine	Ethanol	Endomorphin
	Log2FC, *p* Value	Log2FC, *p* Value	Log2FC, *p* Value	Log2FC, *p* Value	Log2FC, *p* Value	Log2FC, *p* Value
3-Phosphoglyceric Acid		−2.72,0.03	−2.23, 0.04			
Acetylcysteine				−0.74,0.04		
Allantoin		−0.66,0.02		−0.65,0.01		
Creatinine		−0.36,0.01	−0.22, 0.03	−0.27,0.01	−0.23, 0.02	
D-Ribose 5-Phosphate				−1,06, 0.003		
Guanosine Monophosphate					−0.61, 0.005	
Guanosine Triphosphate		Inf,0.04	Inf,0.04	Inf,0.04	Inf,0.04	Inf,0.04
Inosine					−1.19, 0.008	
L-Leucine		−0.26,0.02	−0.17, 0.02		−0.20, 0.01	
L-Methionine		−0.45,0.003	−0.39, 0.02	−0.33,0.01	−0.38, 0.007	−0.27,0.03
L-Phenylalanine	−0.30, 0.003	−0.32,0.007	−0.27, 0.02	−0.25, 0.005	−0.26, 0.002	−0.21,0.04
L-Tyrosine	−0.27, 0.013	−0.30,0.005	−0.22, 0.02	−0.22,0.03	−0.19, 0.0004	
L-Valine	−0.31, 0.03	−0.19,0.04		−0.13,0.04	−0.15, 0.02	
N-Acetylglutamine			−0.85, 0.03			
N-Acetylornithine		−0.51,0.03	−0.56, 0.01		−0.47, 0.04	
Pyruvic Acid		−1.68,0.009				
S-Adenosylhomocysteine			−1.39, 0.04	−1.61,0.01		
Succinic Acid					−0.43, 0.03	

∞/Inf = infinite LC/MS intensity range due to non-detection in the vehicle control group.

All other enviromimetic treatments subsequently exhibited at least 1 specific, and thus unique, metabolome alteration. Ethanol treatment yielded a significant alteration in Succinic Acid (Log2FC = −0.44, *p* = 0.039), Guanosine Monophosphate (Log2FC = −0.62, *p* = 0.0056), and Inosine (Log2FC = −1.2, *p* = 0.0087). Nicotine treatment led to the specific alteration of D-Ribose 5-Phosphate (Log2FC = −1.1, *p* = 0.0037) and Acetylcysteine (Log2FC = −0.75, *p* = 0.04). Both IL17a and cortisol treatment led to the identification of only 1 unique metabolome alteration in each group. Notably, IL17a treatment increased Pyruvic Acid (Log2FC = −1.68, *p* = 0.009), while Cortisol yielded a group specific elevation in N-Acetylglutamine (Log2FC = −0.85, *p* = 0.038). These data thus indicate that, with the exception of WIN 55–212,2 and endomorphin, there were subtle yet selective metabolome alterations present in all other treatment groups.

Of particular note, dorsal forebrain organoids treated with our various enviromimetic treatments tended to exhibit a convergence in differentially regulated metabolites. For example, all treatment groups exhibited a significant differential expression in the expression of L-Phenylalanine in dorsal forebrain organoids. However, L-phenylalanine was not the only common alteration detected between groups. We also detected a significant alteration in the expression of GTP in ethanol, endomorphin, nicotine, IL17a, and cortisol treated organoids. Regulators of GTP and GTP-related signaling were also detected in our Reactome pathway analysis (Fig. 2), and were most notably enriched in nicotine treated human-derived dorsal forebrain organoids (Fig. 2g). A schematic summary of metabolomic convergence and divergence between between groups is schematically provided in Fig. 3e. These data therefore indicate that L-Phenylalanine and GTP expression within ethanol, endomorphin, nicotine, IL17a, and cortisol-treated organoids may reflect a commonly noxious role of these compounds within dorsal forebrain-restricted organoids.

### Single-cell DNA content analysis of enviromimetic treatments within human-derived forebrain organoids

We next considered whether our enviromimetic treatments might elicit an alteration in the proliferative activity and/or cell cycle progression of cells within developing forebrain organoids. Given that our cortical cultures were comprised of ventricular zones enriched for SOX2+ neural stem cells and forebrain-specific FOXG1+ neuronal progenitors (see Fig. 1), our dorsal forebrain organoid cultures are representative of the early stages of corticogenesis whereby ventricular progenitors undergo expansion and amplification for the purpose of supporting neurogenesis and an expanding cortical plate. To yield an unbiased evaluation of cell cycle stage at the whole organoid level, we adapted an established single-cell DNA content analysis [92] that could distinguish cells in G1, S, and G2/M phases. Cells undergoing mitosis characteristically contain increased DNA content due to the DNA replication that occurs as they progress through mitotic cycles. Conversely, cells undergoing apoptosis exhibit fragmented DNA due to the activity of endonucleases that cleave and fragment chromatin into nucleosomal units [93]. Thus, cells undergoing cell division within dorsal forebrain organoids could be identified based on their DNA content [92].

Analysis of single-cell DNA content revealed that there were no substantial alterations in the proportion of G1, S, and G2/M phase cells within human-derived dorsal forebrain organoids that had been treated with IL17a, cortisol, nicotine, ethanol, or endomorphin. However, human-derived dorsal forebrain organoids treated with the cannabinoid agonist WIN 55,212-2 exhibited evidence of increased cell death. Namely, there was a substantial (~2.29 fold) increase in DNA fragmentation within this group, which was co-defined by generally decreased proportions of cells within all other detectable phases of the cell cycle (G1, S, and G2/M phases). Therefore, while we did not identify any alterations in mitotic cell cycle dynamics within our various treatment groups, we did identify a potentially neurotoxic role of the cannabinoid agonist WIN 55,212-2 within human-derived dorsal forebrain organoids.

### Induction of apoptosis and DNA damage by narcotic and neuropsychiatry-related enviromimetic treatments within human-derived forebrain organoids

To further investigate the potential for our narcotic and neuropsychiatric-related enviromimetic treatments to induce apoptosis, we adapted a high-throughput FACS DNA damage and cell death panel to corroborate the results our single-cell DNA content analysis.

**Fig. 4:**
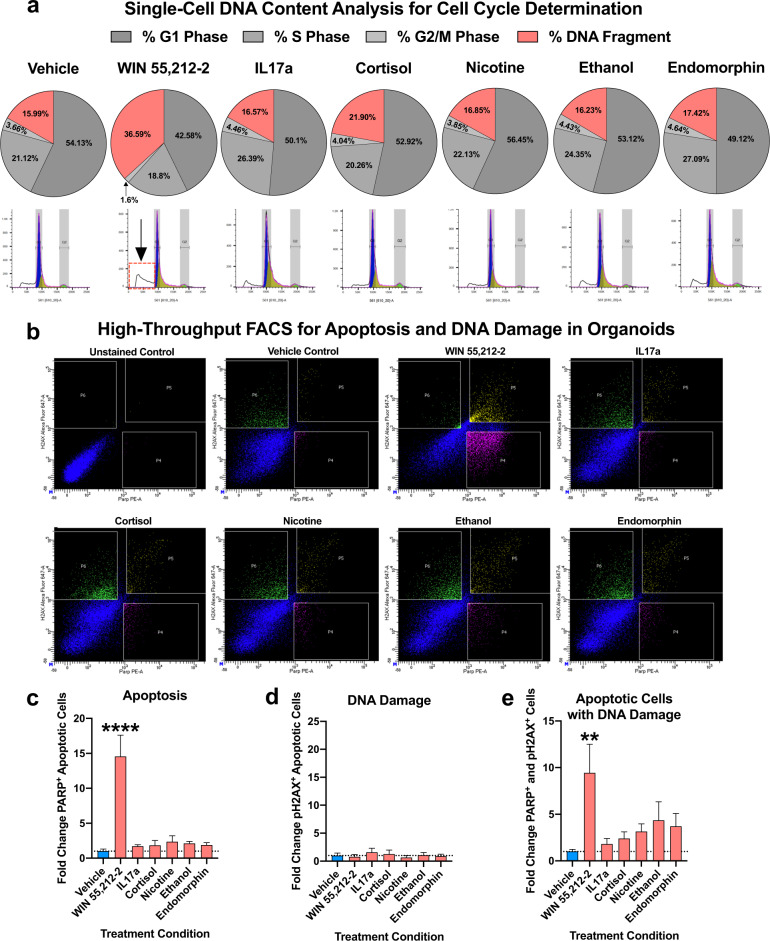
Analysis if DNA content, DNA damage, and apoptosis induction within enviromimetic human-derived forebrain organoids. **a** Increased DNA fragmentation in organoids treated with WIN 55,212-2. To examine if our narcotic and neuropsychiatric-related treatments altered the cell cycle of proliferating cells within human-derived dorsal forebrain organoids, we adapted a single-cell DNA content analysis. Briefly, whole organoids were dissociated to a single-cell suspension and labeled with the fluorescent DNA-intercalating agent Propidium Iodide (PI). DNA content below our G1 peak serves as a proxy of cell death due to DNA fragmentation/digestion by endonucleases [93]. No major alterations in the proportion of proliferating cells or DNA fragmentation were detected in our IL17a, cortisol, nicotine, ethanol, and endomorphin treatment groups. However, a robust effect of WIN 55,212-2 treatment was detected for the DNA content, whereby WIN 55,212-2 selectively increased DNA fragmentation in our human-derived dorsal forebrain organoids. This indicated that, of our various narcotic and neuropsychiatric-related enviromimetic treatments, the cannabinoid agonist WIN 55,212-2 may selectively induce neurotoxicity and thus cell death within developing human-derived dorsal forebrain organoids. DNA histograms are provided for all treatment groups underneath their respective pie-charts, which delineate the proportion of cells in G1 phase, S phase, G2/M phase, and those which exhibit DNA fragmentation (i.e., dead and/or dying cells). For reference, a red-box and arrow identifies the DNA fragmentation peak in dorsal forebrain organoids treated with WIN 55,212-2. **b–e**, Validation of death and DNA damage in organoids treated with WIN 55,212-2. To provide orthogonal validation of our DNA content analysis, we adapted a multi-panel FACS assay to provide a higher-resolution analysis of cell death and DNA damage. Following a series of fixation, permeabilization, and re-fixation steps (designed to gain access to the nucleus), cells were labeled with an Alexa647-conjugated phosphorylated H2AX antibody to detect the presence of DNA damage machinery and a PE-conjugated cleaved PARP antibody for detection of cell death induction. As shown in bar graphs, the endocannabinoid receptor agonist WIN 55,212-2 was the only treatment to significantly increase cell death (PARP-PE + cells, or gate P4 in flow charts) within human-derived dorsal forebrain organoids. There was no increase in P2HAX + healthy cells (i.e., gate P6 in flow charts) that exhibited no evidence of apoptosis amongst any of our treatment groups. However, there was a significant increase in the proportion of DNA damaged apoptotic cells (H2AX + and PARP + double-positive cells, depicted by gate P5 in flow charts) selectively within human-derived dorsal forebrain organoids treated with WIN 55,212-2. For all panels, *n* = 4–6 iPSC donors x *n* = 7 treatment groups for a total *n* of = 34–41 experimental samples for flow cytometry analysis across experiments. All gates/quadrants in flow charts were pseudorandomly labeled. **** denotes *p* < 0.00001 and ** denotes *p* < 0.01. Bar graphs represent mean ± SEM.

To do this, we pseudorandomly selected pools of ~10 organoids per line and condition, and dissociated pools to a single-cell suspension. Cell suspensions were next fixed, permeabilized, resuspended in refixation buffer, and consequently labeled with a PE-conjugated cleaved PARP antibody and Alexa647-conjugated phosphorylated H2AX antibody. Of note, an analysis of unlabeled and labeled samples yielded the unbiased capture and quantification of cells exhibiting cell death and DNA damage within human-derived dorsal forebrain organoids without any background (see unstained flow panel in Fig. 4b). In addition, vehicle-treated controls also exhibited exceptionally low rates of PARP^+^ apoptotic cells (1.58% of all cells), of which only a fraction exhibited evidence of H2AX^+^ DNA damage (0.4% of all cells were double-positive; see top left panel in Fig. 4b). This indicates that dorsal forebrain organoid cultures were broadly healthy, and did not exhibit any evidence of generalized cell death nor DNA damage at baseline.

Analysis of our various enviromimetic dorsal forebrain organoids revealed that all treatment conditions tended to increase cell death. Relative to vehicle-treated controls, the fold change in PARP^+^ cells exhibiting induction of cell death ranged from 1.71 to 14.56 across groups (see Fig. 4a-b). However, ANOVA test statistics indicated that while there was a significant treatment effect, the only drug treatment to significantly elevate apoptosis in dorsal forebrain organoids was the cannabinoid receptor agonist WIN 55,212-2 (*p* < 0.0001). An analysis of the fold change in pH2AX^+^ cells that exhibit DNA damage with or without evidence of apoptosis revealed no significant differences between groups. However, an analysis of the fold change in the proportion of PARP^+^ and pH2AX^+^ cells (which selectively identified DNA damaged cells also undergoing death) revealed a similar outcome to our first analysis. Namely, while all treatment groups tended to exhibit increased proportions of DNA damaged dying cells (fold change ranged from 1.80 to 11.13 relative to controls, across groups), post hoc testing revealed that only WIN 55,212-2 exhibited a statistically significant alteration relative to controls (*p* < 0.01; see Fig. 4d). In sum, only WIN 55,212-2 robustly induced (1) DNA fragmentation, (2) the induction of PARP-mediated cell death, and (3) DNA damage within cells that had already committed to apoptosis.

### Neurogenesis within the developing cortical plate of human-derived forebrain organoids treated with narcotic and neuropsychiatry-related enviromimetics

**Fig. 5:**
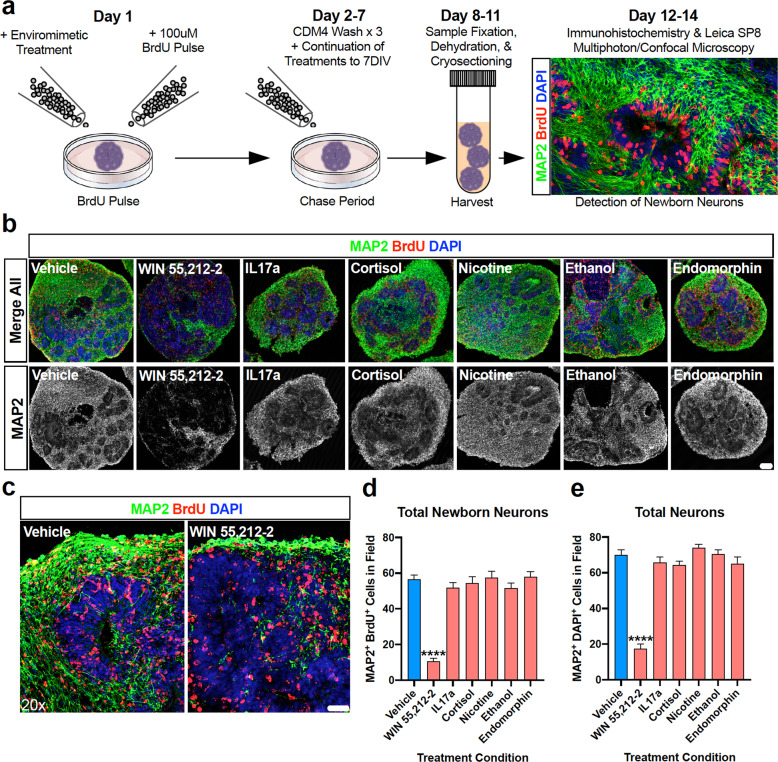
The endocannabinoid agonist WIN 55,212-2 selectively depletes human-derived dorsal forebrain organoids of neurons. **a** Schematic of neocortical neurogenesis pulse-chase assay. Neocortical neurogenesis was evaluated within human-derived dorsal forebrain organoids via the adaptation of a BrdU pulse-chase assay [70]. Briefly, at the time of treatment commencement, dorsal forebrain organoids were pulsed for 24 h with BrdU. Following this, BrdU was removed and washed out and cultures underwent continuation of their allotted treatment to their endpoints. Organoids were subsequently processed (fixed, dehydrated, cryosectioned, mounted) before being immunostained and imaged for newborn (BrdU + ; red) neurons (MAP2 + ; green) via laser-scanning confocal microscopy. **b** Representative whole organoid images of enviromimetic treated organoids. Representative whole organoid images for each treatment group are shown as insets. Note that in all groups there was widespread and robust expression of both BrdU (red) and MAP2 (green) across the entire organoid, except those treated with WIN 55,212-2. To emphasize this, MAP2-isolated channels are presented in gray scale below merged images. **c**–**e** Depletion of newborn and total neurons in WIN 55,212-2 treated organoids. To determine if our various treatments influenced neuron numbers, we quantified the number of newborn neurons MAP2^+^ green cells with BrdU^+^ red nuclei, see (**d**) and total neurons MAP2^+^ green cells with DAPI^+^ blue nuclei, see (**e**) within cortical fields. The only group to exhibit a significant difference in both newborn and total neuron numbers within the developing cortical plates of dorsal forebrain organoids was the cannabinoid agonist WIN 55,212-2. More specifically, we identified that WIN 55,212-2 treatment reduced newborn neurons by 81.23% and total neurons by 75%. When combined with our single-cell DNA content and DNA-damage cell death flow cytometry panels presented in Fig. 4, this data cumulatively confirms that WIN 55,212-2 is acutely neurotoxic and developmentally disruptive within human-derived dorsal forebrain organoids. For all panels, *n* = 3 iPSC donors x *n* = 7 treatment groups x *n* = 3–4 organoid replicates per donor/condition yielded a total *n* of = 18–22 cortical fields per condition. **** denotes *p* < 0.0001. Bar graphs represent mean ± SEM. Scale bar in *b* = 100 μm and c = 40 μm.

During early forebrain development, ventricular progenitors proliferate, differentiate into newborn neurons, and begin their migration from the ventricular zone into the developing cortical plate [94]. This process is both present and conserved within our 3D human-derived dorsal forebrain organoids (see Fig. 1b). Given validation that the cannabinoid agonist WIN 55,212-2 selectively exerted neurotoxic effects (Fig. 4) in human-derived dorsal forebrain organoids (Fig. 4), we next sought to examine whether WIN 55,212-2 and our other narcotic and neuropsychiatric-related treatments altered neuron numbers within our human-derived dorsal forebrain organoid system. To do this, we adapted a BrdU pulse-chase assay to examine neocortical neurogenesis within organoids [70]. Briefly, at the commencement of drug treatment, organoids were pulsed with 100 μM BrdU for 24 h before continuing their compound exposure routines for a 7 DIV chase period (see Fig. 5a for schematic). This protocol therefore enabled us to label progenitors that were specifically differentiating at the time of drug exposure and track the total quantities of newborn neurons that were subsequently generated. This approach therefore controls for baseline differences in neuron numbers within each organoid, as well as ensures that a specific effect of each drug compound can be identified and assessed for potential effects upon neurogenesis. Analysis revealed that treatment with IL17a, cortisol, nicotine, ethanol, and endomorphin elicited a lack of effect on newborn and total neuron numbers within the developing cortical fields of human-derived dorsal forebrain organoids. Contrary to this, organoids treated with WIN 55,212-2 exhibited a robust depletion of both newborn neurons (MAP2 + neurons with BrdU+ nuclei) and total neurons (MAP2 + neurons with DAPI + nuclei). This indicates that the cannabinoid CB_1_ agonist WIN 55,212-2 interferes with neocortical neurogenesis within developing human-derived forebrain tissue. This confirms that WIN 55,212-2, and ergo cannabinoids, are potentially noxious compounds during early human fetal brain development.

## Discussion

The current study sought to determine prenatal signatures related to narcotic use and mental illnesses within 3D human-derived forebrain tissue. In experiments conducted in parallel, we modeled exposure to opiates (μ-opioid agonist endomorphin), cannabinoids (WIN 55,212-2), alcohol (ethanol), smoking (nicotine), chronic stress (human cortisol), and maternal immune activation (human IL17a). Following a range of high-throughput assays, we identified both convergent and divergent signatures between enviromimetic treatment groups, and unbiasedly identified the cannabinoid agonist WIN 55,212-2 as a particularly noxious agent that severely impacts normative cortical development within 3D human-derived dorsal forebrain organoids.

Our first goal was to establish the macromolecular effects of our enviromimetic treatments within human-derived dorsal forebrain organoids. We identified that the molecular composition of enviromimetic-treated organoids were relatively similar, however mapping differentially expressed proteins into their respective Reactome pathways provided additional clarity. Notably, IL17a identified alterations in signaling by ERBB2 factors, which is a pathway of particular relevance to schizophrenia given its interaction with neuregulin-1 to mediate cell adhesion [95], its potential role in schizophrenia risk [96], as well as antipsychotic response/treatment [97]. This result is also of note as MIA experiments in rodents have also identified neuregulin-1 and EGF-related (notably, ERBB4) differences in offspring [98, 99]. Similarly, we also identified WNT signaling dysfunction in our nicotine treated organoids, which is broadly important in the context of neurodevelopmental programming [100, 101], autism risk [102, 103], and schizophrenia too [104–106]. Recent work has shown that nicotine alters embryonic stem cell proliferation [107], and it has been suggested that nicotine may thus also alter neural progenitors as well as neurogenesis [108]. Given the prominence of WNT signaling in the developing brain, these data only reinforce the idea that prenatal exposure to nicotine may have the potential to elicit neurodevelopmental alterations. Nicotine and ethanol treated organoids also mutually exhibited enriched pathways for brain development, including factors related to axon guidance, ROBO receptor signaling, as well as mRNA metabolism and mRNA regulatory pathways. This is prominent as ROBO receptors play an evolutionarily conserved role in both short- and long-distance axon path finding and guidance [109–111]. Similar to nicotine and ethanol, organoids treated with WIN 55,212-2 and endomorphin treatment also exhibited enrichment for mRNA regulatory processes including degradation by 5ʹ to 3ʹ endonucleases and EJC-mediated Nonsense Mediated Decay (NMD), respectively. These pathways, such as NMD, function as important quality and quantity control mechanisms of gene expression [112], and their enrichment within our datasets collectively suggest that there may be disparate, yet similar, responses of these drug treatments upon mRNA degradation and RNA regulatory pathways within the developing brain. However, little work has been conducted on how these fundamental mRNA degradation pathways may regulate neurodevelopment; although it is likely that they are to play an essential role during neural development [113]. The NMD machinery has been implicated in both neural progenitor activity [113] and neuronal differentiation [114]. Notably, NMD has also been shown to specifically regulate axonal guidance in ex vivo open book preparations [115]. Furthermore, in recent work, we have shown that the NMD machinery is expressed in neurons and is operational within their dendrites, where NMD functions to regulate GLUR1 expression, LTP, as well as learning and memory [116]. Variants within the NMD machinery, notably UPF3, have also been associated with developmental delay [117], mental retardation [118], and childhood onset schizophrenia [119]. Therefore, data from our nicotine, ethanol, endomorphin, and WIN 55,212-2 groups indicates that RNA regulation is likely to play a role in the pathogenic effects of these treatments within developing human tissue.

To provide further systems-level analysis of our enviromimetic treatments in human-derived dorsal forebrain organoids, we adapted hybrid MS to globally map the developing organoid metabolome. This led to the observation that several factors exhibited convergent alterations in all groups, notably L-Phenylalanine. Prior work has indicated that Phylalanine adversely affects the developing mammalian brain [120] via a variety of mechanisms. For instance, phenylalanine has been shown to induce neuronal death [121], potentiate oxidative stress in the developing cerebral cortex of rats [122], and alter the acid-soluble pool of particular amino acids which modulates their incorporation into proteins within the rat brain [123]. Consistent with this, in Phenylketonuria—a disease characterized by elevated levels of pheylalanine in plasma and cerebrospinal fluid of patients—there are delays in brain development that yields profound insufficiencies in cognitive performance [121]. Thus, the differential expression of L-Phenylalanine in all treatment groups relative to controls indicates a potentially noxious role of all compounds studied within developing dorsal forebrain organoids. This is consistent with discrete alterations in amino acid expression across groups (L-Tyrosine and L-Valine in WIN 55,212-2 treated organoids, and in all other groups L-Methionine).

Excluding WIN, 55,212-2, all other treatment groups also exhibited an alteration in the expression of GTP, which is essential for normative cortical development. Similar to phenylalanine, metabolomic alterations in GTP are also directly linked to neurological disease; namely Segawa disease, which is characterized by deficient dopamine content and dopa-responsive dystonia [124]. However, GTP is broadly more important within the brain, as hydrolysis of GTP into 7,8-DHNP-3’-TP is required for the biosynthesis of numerous monoamine neurotransmitters. Therefore, an intrinsic alteration in GTP within our enviromimetic dorsal forebrain organoids is also likely to hold important implications for healthy corticogenesis within the developing forebrain of our human-derived organoids. In support of this, neuronal GTPase activators are ubiquitously expressed during cortical development [125]. In addition, Rho GTPase proteins [126] and signaling [127] are also known to specifically regulate progenitor proliferation and survival (e.g., RAC1) [128], cell fate of neural progenitors (e.g., CDC42) [129], neuronal migration [126, 127], and axonal development [130]. Therefore, GTP is likely to be important during all phases of brain development and maturation as GTPase factors regulate neural progenitors as well as neuronal differentiation and maturation. Therefore, similar to L-Phenylalanine, significant differences in GTP expression within ethanol, endomorphin, nicotine, IL17a, and cortisol-treated organoids likely reflects a commonly noxious nature of these compounds during human corticogenesis.

Aside from these common metabolic signatures, we also identified a number of highly specific group differences. First, we identified that pyruvic acid was selectively altered in IL17a treated organoids. Interestingly, a prior study has shown that targeting of purine pathway metabolites may be effective in treating autism-related phenotypes in a MIA mouse model [131]. This is paralleled in work that is indicative that pyruvic acid may be abnormal in autism spectrum disorders and may serve as a potential biomarker of the disease and/or a potential therapeutic target [132, 133]. In addition, purine/pyramidine metabolites have been broadly associated with a range of neuropsychiatric disorders that are also defined by neurodevelopmental risk [134]. This further supports the idea that this factor may be a useful biomarker for neuropsychiatric sequelae that may arise from maternal immune activation. Likewise, we identified N-acetylglutamine—which is the acetylated analog of glutamine—as a specific marker of chronic CORT exposure in human-derived dorsal forebrain organoids. This reflects a novel association that does not appear to have been reported in prior works, and little is known of N-acetylglutamine in the developing brain in general. However, given the role of N-acetylglutamine and glutamine in the synthesis of numerous essential units within the cell (e.g., proteins) [135], this result is likely to hold functional importance and will, accordingly, require further investigation. In our ethanol treated organoids, two unique metabolites were identified. The first, succinic acid, may be reflective of generalized alcohol exposure due to the formation of this metabolite during alcoholic fermentation [136], but is a relatively novel hit considering a lack of studies on the topic. The second, Guanosine Monophosphate Inosine, has been identified in a pathway analysis screening of metabolite dysfunction in an animal model of alcoholic liver disease [137] but otherwise also remains a novel hit. Further work on these two potential corollaries may therefore unveil their biological importance in mediating the effects of prenatal alcohol exposure on the developing brain. Last, our analysis of nicotine treated organoids returned group-specific alterations in D-Ribose 5-Phosphate and Acetylcysteine. D-Ribose 5-Phosphate has been previously identified within tobacco leaves [138] but has been scarcely studied, especially in the context of it being a metabolite marker of neuropathology in progenitors, neurons, and brain development. On the other hand, Acetylcysteine is amongst the most widely studied metabolite factors in neuropsychiatry due to its antioxidant properties, and has been extensively studied for its use in the management of substance use disorders [139]. This includes cue-induced nicotine seeking as an endophenotype of smoking behavior in rodents [140–142] well as nicotine-dependence and tobacco use disorder within humans [143, 144]. Acetylcysteine has also been extensively studied and shown to have potential therapeutic benefits in both patient-derived iPSC [145] and a glutathione-deficient rodent model of Schizophrenia [146]. As an estimated ~10.7% of mothers whom smoke will continue to do so during pregnancy, and considering evidence that nicotine may elicit prenatal effects on the developing brain (see [21]), it is imperative that further studies follow-up the metabolite markers here for their role as potential biomarkers as well as their utility as potential therapeutic targets.

We also identified that a variety of our narcotic and enviromimetic organoids also exhibited Reactome pathway alterations related to cell-cycle checkpoints/regulation, mitosis, cellular stress response pathways, as well as apoptosis and the apoptosome (see Fig. 2). Given the importance of progenitor proliferation and survival in the developing brain, these data consequently led us to next consider the impact of our treatments on cell-cycle activity and cell death. This revealed that there were no major differences in the proportion of cells in G1, S, or G2/M phases of the cell cycle between groups. The exception to this was human-derived organoids treated with the cannabinoid agonist WIN 55,212-2, which exhibited alterations due to a dramatic increase in DNA fragmentation (Fig. 3a). DNA fragmentation occurs when endonucleases cleave chromatin into nucleosomal units, and is therefore a marker of cells undergoing apoptosis [93]. Consistent with this, in a follow-up flow cytometry panel we identified that organoids treated with WIN 55,212-2 were selectively enriched for cells exhibiting both apoptosis (cleaved PARP) and DNA damage machinery (pH2AX, see Fig. 3b–e). Importantly, cells which exhibit minor DNA damage will attempt to repair such, whereas those that exhibit more DNA damage will be targeted for apoptosis [147]. The increase in both apoptotic cells, and the proportion of apoptotic cells exhibiting DNA damage, in WIN 55,212-2 treated forebrain organoids is therefore indicative of acute neurotoxicity. Furthermore, in our neocortical neurogenesis pulse-chase assay, WIN 55,212-2 was also the only mimetic condition to elicit a robust depletion in both total neuron and newborn neuron numbers (Fig. 5). This cross-validation therefore confirms that WIN 55,212-2 is acutely noxious in human-derived dorsal forebrain organoids. This result is important, as it addresses and contributes to a major point of contention within the literature. Notably, while there is consensus that prenatal cannabinoid exposure likely alters human neuron development [148] and function [149] in a manner that likely induces protracted risk to behavioral alterations [150, 151], there is conflicting data regarding the specificity of cannabinoid use/abuse/stimulation/modeling upon cell death. For example, synthetic cannabinoids (the accessibility and abuse of which have become a major public health concern [148]) have been shown to elicit cytotoxic effects via the CB_1_ receptor in mouse forebrain cultures [152]. However, there is also a rich literature which indicates that cannabinoids may also be able to elicit neuroprotective effects by preventing apoptosis [153]. Contributing further ambiguity, at least one study reported that the treatment of pregnant dames with WIN 55,212-2 did not alter cell death within the prenatal mouse cerebral cortex [154]. Indeed, these conflicting data are well described in the literature, and it has been proposed that cannabinoids may elicit both neuroprotective and pro-apoptotic effects in a non-binary and therefore sophisticated manner [155]. Cannabinoid effects on cell death may therefore be dependent upon the cell-type target and the developmental stage at which exposure occurred [156]. Yet, in our prenatal model of the developing forebrain, we observed reproducible evidence of cell death in multiple single-cell analyses (Fig. 4) that were accompanied by a dramatic decrease in neocortical neurogenesis (Fig. 5). These data may therefore be indicative of a potential ‘species-of-origin’ effect, or indicate that other experimental considerations may mediate neurotoxic effects.

It is therefore important to emphasize that the mechanisms of action of synthetic cannabinoids may differ from plant-derived compounds, and that differing concentrations, frequency of use, and timing of consumption may all elicit differential effects. A limitation of this panel study is therefore the fact that only one concentration of each factor was studied, at one particular early time point of development that coincided with early corticogenesis approximately equivalent to trimester one. Effects may therefore scale with increasing doses, longer durations of exposure, or later developmental time points. In addition, it is important to note that while cerebral organoids reproduce aspects of fetal brain transcription [67], epigenetic regulation [68, 69], and proteomic programing [70], this model is not a perfect substitute for the developing brain and yet other factors, phenotypes, or mechanisms may exist outside of those identified in the current study. However, given rising rates of cannabinoid use (both plant-based and synthetic derivatives), such experimental considerations and limitations are important to explicate so not to unnecessarily stigmatize recreational users in jurisdictions where legalization has occurred and/or is currently unfolding. Nonetheless, our work still provides an important contribution to the literature by indicating that chronic WIN 55,212-2 exposure elicits a robust and reproducible neurotoxic effect within human-derived developing forebrain tissue that signals a need for caution. This work therefore emphasizes the need for yet more cannabinoid effects to be reevaluated specifically within human-derived cells and 3D tissue systems, as well as warrants independent replication and further study by other groups.

Following from the work presented here, it will be important for a number of future directions to be explored in more extensive, as well as more specific and topical, investigations. For example, it is possible that other subtle phenotypes may exist in WIN 55,212-2, as well as other, treatment groups. For example, it is of specific note that the endocannabinoid system has been shown to have a potential role in astroglial cell phenotypes, including their differentiation [157]. Indeed, cerebral organoids have already been used to study glial cell diversity and methamphetamine-induced neuroinflammation [158], indicating that similar work could be completed by substituting cannabinoids as well as other agents. Therefore, a future direction following from this work may therefore be to consider differences in the production and proportion of glia, such as astrocytes, in future drug panel organoid studies. In addition, it will be equally important to determine how different types of neurons may be impacted by the enviromimetic conditions examined here. Likewise, it will also be important to continue this line of investigation by examining neuronal dynamics and activity differences. Therefore, a fruitful avenue of further research may be to design a physiological panel to examine how our various treatment groups alter neuronal electrophysiology via high-throughput approaches. Conceptually, due to the overlapping nature of comorbidities and the fact that numerous substances may be simultaneously abused, future studies may wish to consider the compounding effects of simultaneous risk factor exposure within this dorsal forebrain organoid system. Lastly, arguably the most important future direction to emerge from this work would be for other groups to independently replicate our findings, and to adapt the specific controlled and scheduled substances that were modeled here with established mimetic compounds.

In closing, the current study supports the idea that prenatal exposure to a variety of factors can alter the proteome, metabolome, and other discrete cellular processes that are essential for normative cortical development in 3D human-derived developing forebrain tissue. This multi-omic pipeline therefore provides important insight into the dynamics of prenatal brain development, and serves as a valuable human tissue resource that can be screened and cross-referenced by preclinical and clinical researchers alike to identify factors that may mediate the prenatal effects of various drug and neuropsychiatric risk factors in their own datasets.

## Materials and methods

### Human induced pluripotent stem cells

Human Induced Pluripotent Stem Cells (iPSCs) were maintained as previously described (see [70]). Briefly, iPSCs were maintained in vitronectin-coated plates and passaged using Accutase (Sigma, Material#: A6964) for progression to cerebral organoid generation or with EDTA (prepared in house) for expansion and maintenance. Cells were fed every 24–48 h, as necessary, with mTeSR Plus media (Stem Cell Technologies, Material#: 05825). Human iPSC lines were cultured simultaneously to control for idiosyncratic culturing conditions. All human iPSCs were acquired from deposits made to the National Institute of Mental Health (NIMH) Repository & Genomics Resource center at Rutger’s University (lines beginning with prefix MH, found below) or from the Coriell Institute for Medical Research (lines beginning with prefix GM, also found below). Thus, lines had typically undergone extensive, standardized, testing for common iPSC factors such as pluripotency, viability, and karyotypes. In total, 6 different iPSC lines were sampled across experiments included in the manuscript (MH0159019, MH0159020, MH0159022, MH0174677, GM23279, GM25256) with tissue originating from apparently healthy individuals without any neuropsychiatric or neurological diagnosis or evidence of family history of such. The patients are otherwise listed as healthy donors and devoid of any conflicting or confounding diagnoses. Healthy patient donors comprised 4 males (MH0159020, MH0159022, MH0174677, GM25256) and 2 females (MH0159019 and GM23279). No sex difference in phenotype was observed between iPSC lines. All but one of the donors were adults at the time of biopsy (range: 9, 29, 30, 36, 46, and 58 years of age), alas no age-mediated differences between the iPSC lines were identified in any experimental assay or in quality control assessments.

### Generation of human-derived dorsal forebrain organoids

Dorsal forebrain organoids were derived via a directed differentiation protocol from Paola Arlotta’s laboratory at Harvard [71] that we have amended for an expedited/shorter timeline as previously described [70]. Briefly, we cultured undifferentiated iPSCs into colonies before dissociating these into a single cell suspension via Accutase exposure (see above). Suspensions were correspondingly transferred and cultured within ultra-low attachment Aggrewell V-Bottom Plates (Stem Cell Tech, CAT#: 34815) so that 3D stem cell aggregates known as embryoid bodies could be formed. Because of the dimensions and shape of wells within Aggrewell plates, embryoid bodies were reproducibly formed into spherical geometries of a consistent size both within and between iPSC lines and batches. From this point, 3D tissue samples were cycled through successive Cortical Differentiation Media (CDM1–4) every ~7–10 days for a final timeline that yielded early cortical organoids by ~30 DIV. CDM chemical components are described at length within [71]. By the end of our culturing period, all organoids exhibited robust induction of expected morphologies (e.g., ventricles surrounded by ventricular zones) as well as forebrain-specific markers (e.g., FOXG1 + cells) and cortical-specific neurons (e.g. CTIP2 + cells; see Fig. 1). For more extensive protocol details, please refer to [71] for reagents and [70] for timing and other design considerations.

### Treatment regime

Human-derived dorsal forebrain organoids were systemically exposed to a barrage of widely accepted drug and environmental mimetics, in tandem, for a total of 7 Days In Vitro (7 DIV). This ensured that all control and treatment conditions were cultured in parallel simultaneously under exacted laboratory conditions (e.g., media batches, feeding regimes etc.). Concentrations were utilized that were known to be phenotypic but not noxious to cultures and/or animals. Consequently, all concentrations were derived from prior studies that generally did not report major neurotoxic effects of each compound, thus avoiding potential ceiling effects on phenotypes. Thus, concentrations were adapted principally from prior basic mechanism studies (and, in our own testing pre-experiment testing, organoid survival) as this allowed a degree of methodological standardization and precision across our 7 different groups that would have not been otherwise achievable. To model alcohol exposure, we utilized a 100 mM [159] of molecular biology grade ethanol (Fisher Scientific, Material#: BP2818100). This concentration is consistent with concentrations reported for ethanol to effect normative brain function in naïve and occasional users [160]. To model chronic stress, we utilized a 10 μg/mL concentration [161] of the human stress hormone cortisol (CORT). We specifically utilized a water-soluble version of CORT, namely hydrocortisone-hemisuccinate (Sigma, Material#: H2270). Similar to ethanol, CORT treatment is a common method for modeling chronic stress under controlled laboratory conditions in both slice [161] and animal studies [60, 162–164]. Cannabis exposure was modeled using a 10 μM concentration [165] of the cannabinoid receptor agonist WIN 55,212-2 (Sigma, Material#: W102), which has also been widely used in cell [166] brain slice [165, 167] studies. To model maternal immune activation, we treated developing dorsal forebrain organoids with 10 ng/mL [168–170] of the human cytokine IL17a (Sigma, Material#: H7791). Importantly, IL17a is downstream of IL6 and was recently shown to mediate the brain-specific effects of maternal immune activation [54, 171]. Therefore, IL17a maintains the necessary construct and predictive validity required to model immune exposure in tissue that otherwise lacks an endogenous immune system. Opiate use was modeled utilizing the μ-opioid receptor agonist endomorphin-1 (1 μM, Abcam, Material#: ab1240411) as utilized in prior studies [172–174]. Last, to model prenatal exposure to smoking, we treated organoids with the nicotinic acetylcholine receptor agonist (-)-nicotine ditartrate (10 μM, Abcam, Material#: ab120562) which was similar to concentrations utilized in prior studies too [175–177]. All concentrations adapted were screened to ensure that no overt evidence of organoid deterioration was induced via chronic exposure for 7 DIV, thus avoiding any manifest ceiling effects upon cell viability and/or death.

### Unbiased flow cytometry of apoptosis & DNA damage

To derive an unbiased assessment of DNA damage, cell death, and DNA-damaged cells undergoing apoptosis following drug treatment, we adapted a well validated and widely utilized a Fluorescent-Activated Flow Cytometry (FACS) kit (BD Pharmingen, Material#: 562253). Briefly, pseudorandomly selected organoids were dissociated to a single-cell suspension via a 20-minute exposure to accutase followed by tricheration and serial filtering through 70 → 30 μm pores. Cells were resuspended and incubated in in Cytofix/Cytoperm solution for fixation and initial permeabilization for 30-minutes at room temperature. Cells were consequently washed, resuspended in BD “Plus” permeabilization buffer for 10-minutes on ice, and re-exposed to Cytofix/Cytoperm solution for 5 additional minutes to achieve refixation. Cells were consequently washed, and resuspended in 30 μg of DNAse for 60-minutes at 37 °C. Cells were consequently washed and labeled with PE-Cleaved PARP (BD Pharmingen, Material#: 51–9007684) to label cells committed to apoptosis and Alexa647-H2AX (BD Pharmingen, Material#: 51–9007683) for cells exhibiting DNA damage. Double positive cells represented apoptotic cells exhibiting DNA damage. Per manufacturer instructions, antibodies were diluted at a 1:25 ratio and incubated for 20-minutes at room temperature. Cells were washed and resuspended in staining solution. Labeled suspensions were analyzed utilizing a BD Aria II (Becton Dickinson) cell sorter to acquire multiparameter data files. Data were presented as fold-change of % of cleaved PARP + apoptotic cells, % of H2AX + DNA damaged cells, and % of PARP + H2AX + double-positive cells.

### Cellular DNA content & cell-cycle analysis

Organoids were prepared to a single-cell suspension as described above and in [70], and washed successively with calcium/magnesium free PBS at 4 °C to remove residual peptides in solution. Samples were consequently centrifuged, supernatant removed, and pelleted cells resuspended in PBS. Cells were EtOH fixed (100%, at 4 °C) while being gently vortexed. Cells were rehydrated, and incubated with Triton-X with DNAse added for 5 min. Cells were pelleted, Triton-X removed, and resuspended in 200ul of calcium/magnesium free PBS at 4 °C. Immediately prior to flow cytometry, suspensions were incubated with 2 μL of Propidium Iodide (PI; Thermofisher, Material#: P3566), and analyzed for single-cell DNA content utilizing a Sony MA900 cell analysis cytometer. Cell-cycle was subsequently modeled post hoc in the FloJo cytometry analysis package (Becton Dickinson), which allowed cells in G1, S, and G2/M phases to be distinguished based on the DNA content of each individual captured cell.

### Immunohistochemistry and laser-scanning confocal microscopy

Immunohistochemistry of organoids was conducted as previously described [70]. Briefly, organoids were drop-fixed in 4% paraformaldehyde, dehydrated in 30% sucrose, embedded in (Tissue Tek, Material#: 4583) using biopsy molds, and cryosectioned at 30 µm. All sections underwent antigen retrieval in citrate buffer, and were incubated in primary overnight. Primary antibodies comprised SOX2 (1:1000; R&D Systems, Material#: MAB2018-SP), TUJ1/β-tubulin III (1:1000; Abcam, Material#: AB41489), MAP2 (1:1000, Abcam, Material#: AB11267; 1:1000, Abcam, Material#: AB32454), BrdU (1:1000, BD Pharmingen, Material#: 555627), CTIP2 (1:300; Abcam, Material#: AB18465), and FOXG1 (1:500, Abcam, Material#: ab18259). Secondary antibodies were incubated for 2 h at room temperature, and comprised antibodies for rabbit (Fluor 488 Material#: A11008; Fluor 546 Material#: A11035; & Fluor 633 Material#: A21070), mouse (Fluor 488 Material#: A11001; Fluor 546 Material#: A11003; & Fluor 633 Material#: A21052) and chicken (Fluor 546 Material#: A11040). All secondary antibodies were used at a 1:2000 dilution, and sourced from Life Technologies. Microscopy was completed on an Olympus IX81 Laser-Scanning Confocal Microscope or Leica SP8 Multiphoton/Confocal microscope. Images were typically acquired at 1200 × 1200 resolution with optical *Z* slices (step sizes) ranging from 0.5 to 10 µm depending on the unit of analysis.

### Neocortical neurogenesis pulse-chase paradigm

To examine neurogenesis, we conducted a 7DIV BrdU pulse-chase experiment in dorsal forebrain organoids as previously described [70]. Briefly, organoids were pulsed with 10 μM BrdU for 24 h time-locked to when drug treatment commenced. This yielded widespread BrdU incorporation into all proliferating and/or differentiating cells within organoids over a 24 h window. Following this, organoids were maintained for 7DIV with or without treatment, at which point organoids were drop fixed in 4% PFA, dehydrated via sucrose incubation, embedded using Tissue-Tek OCT (CAT#: 4583) compound at −80 °C, and subsequently cryosectioned. Analysis involved immunohistochemistry for new-born cells that exhibit BrdU + (1:1000, BD Pharmingen, CAT#: 555627) nuclei within MAP2 + cell bodies. This subsequently allowed interpretation of the relative degree to which cells that were proliferating at the commencement of our enviromimetic treatments underwent terminal differentiation into neurons (BrdU^+^ MAP2^+^ double-positive cells).

### Proteomics, barcoding chemistry, & liquid-chromatography/mass-spectrometry

Tandem Mass Tag (TMT) Liquid-Chromatography/Mass-Spectrometry (LC/MS) proteomics was completed as previously described in [70] and [178]. Briefly, organoids were reduced with dithiotreitol and underwent alkylation with iodoacetamide before tryptic digestion at 37 °C overnight. Peptide suspensions were desalted using C18 stage-tips prior to Liquid Chromatography-Mass Spectometry (LC-MS) analysis. An EASY-nLC 1200, which was coupled to a Fusion Lumos mass spectrometer, (ThermoFisher Scientific) was utilized. Buffer A (0.1% FA in water) and buffer B (0.1% FA in 80% ACN) were used as mobile phases for gradient separation [178]. A 75 µm I.D. column (ReproSil-Pur C18-AQ, 3 µm, Dr. Maisch GmbH, German) was packed in-house for separating peptides. A separation gradient of 5–10% buffer B over 1 min, 10–35% buffer B over 229 min, and 35–100% B over 5 min at a flow rate of 300nL/min was adapted. Data dependent mode was selected during operation of the Fusion Lumos mass spectrometer. An Orbitrap mass analyzer acquired Full MS scans over a range of 350–1500 m/z with resolution 120,000 at m/z 200. The top 20 most-abundant precursors were selected with an isolation window of 0.7 Thomsons and fragmented by higher-energy collisional dissociation with normalized collision energy of 40. The Orbitrap mass analyzer was also used to acquire MS/MS scans. The automatic gain control target value was 1e6 for full scans and 5e4 for MS/MS scans respectively, and the maximum ion injection time was 54 ms for both. For TMT chemistry, we adapted the recently released TMTpro 16Plex labeling reagents from ThermoFisher Scientific. This allowed us to run 28 total samples for MS detection of proteins, not including pools for internal standardization, allowing us to sample all 7 group conditions from organoids generated from a maximum of 4 independent iPSC lines (iPSCs ending in 020, 022, 279, and 677 were randomly selected for TMT-LC/MS).

### Bioinformatics for proteomics

MS raw files were analyzed using the MaxQuant software [179] and peptide lists were searched against the human Uniprot FASTA database with the Andromeda search engine [180]. A contaminants database was employed and cysteine carbamidomethylation was set as a fixed modification and N-terminal acetylation and methionine oxidations as variable modifications. Further modifications included TMT tags on peptide N termini/lysine residues (+229.16293 Da) set as static modifications. False discovery rate (FDR) was 0.01 for both the protein and peptide level with a minimum length of 7 amino acids for peptides and this FDR was determined by searching a reverse sequence database. Enzyme specificity was set as C-terminal to arginine and lysine as expected using trypsin protease, and a maximum of two missed cleavages were allowed. Peptides were identified with an initial precursor mass deviation of up to 7 ppm and a fragment mass deviation of 20 ppm. Protein identification required at least one unique or razor peptide per protein group. Contaminants, and reverse identification were excluded from further data analysis. Protein intensities were log2 transformed and normalized using quantile normalization from R package preprocessCore. The histogram of the precursor intensity distribution and the boxplot of correlation covariance were visualized using R package ggplot2. Proteins with no missing values were subjected to downstream visualization and statistical analysis using Perseus software of the MaxQuant computational platform [181]. Proteins were subjected to one-way ANOVA test followed by post-hoc test. Proteins with *p* < 0.05 in one-way ANOVA and differentially regulated versus vehicle controls in post-hoc test were included in downstream functional enrichment analysis. STRING and Reactome databases were utilized for functional enrichment and pathway analysis with significance at *p* < 0.05.

### Hybrid metabolomics liquid-chromatography/mass-spectrometry

Randomly selected samples (organoids generated from iPSCs ending in 019, 020, 022, 256, and 677, each weighing ~20 mg) were subjected to an LC/MS analysis to detect and quantify known peaks. A metabolite extraction was carried out on each sample based on a previously described method [182]. The LC column was a Millipore^TM^ ZIC-pHILIC (2.1 ×150 mm, 5 μm) coupled to a Dionex Ultimate 3000^TM^ system and the column oven temperature was set to 25^o^C for the gradient elution. A flow rate of 100 μL/min was used with the following buffers; (A) 10 mM ammonium carbonate in water, pH 9.0, and (B) neat acetonitrile. The gradient profile was as follows; 80–20%B (0–30 min), 20–80%B (30–31 min), 80–80%B (31–42 min). Injection volume was set to 2 μL for all analyses (42 min total run time per injection). MS analyses were carried out by coupling the LC system to a Thermo Q Exactive HF^TM^ mass spectrometer operating in heated electrospray ionization mode (HESI). Method duration was 30 min with a polarity switching data-dependent Top 5 method for both positive and negative modes. Spray voltage for both positive and negative modes was 3.5 kV and capillary temperature was set to 320^o^C with a sheath gas rate of 35, aux gas of 10, and max spray current of 100 μA. The full MS scan for both polarities utilized 120,000 resolution with an AGC target of 3e6 and a maximum IT of 100 ms, and the scan range was from 67–1000 *m*/*z*. Tandem MS spectra for both positive and negative mode used a resolution of 15,000, AGC target of 1e5, maximum IT of 50 ms, isolation window of 0.4 m/z, isolation offset of 0.1 m/z, fixed first mass of 50 m/z, and 3-way multiplexed normalized collision energies (nCE) of 10, 35, 80. The minimum AGC target was 1e4 with an intensity threshold of 2e5. All data were acquired in profile mode.

### Bioinformatics for metabolomics

The resulting Thermo^TM^ RAW files were converted to mzXML format using ReAdW.exe version 4.3.1 to enable peak detection and quantification. The centroided data were searched using an in-house python script Mighty_skeleton version 0.0.2 and peak heights were extracted from the mzXML files based on a previously established library of metabolite retention times and accurate masses adapted from the Whitehead Institute [183], and verified with authentic standards and/or high resolution MS/MS spectral manually curated against the NIST14MS/MS [184] and METLIN [185] tandem mass spectral libraries. Metabolite peaks were extracted based on the theoretical *m*/*z* of the expected ion type e.g., [M + H] + , with a ± 5 part-per-million (ppm) tolerance, and a ± 7.5 s peak apex retention time tolerance within an initial retention time search window of ± 0.5 min across the study samples. The resulting data matrix of metabolite intensities for all samples and blank controls was processed with an in-house statistical pipeline Metabolyze version 1.0 and final peak detection was calculated based on a signal to noise ratio (S/N) of 3X compared to blank controls, with a floor of 10,000 (arbitrary units). For samples where the peak intensity was lower than the blank threshold, metabolites were annotated as not detected, and the threshold value was imputed for any statistical comparisons to enable an estimate of the fold change as applicable. The resulting blank corrected data matrix was then used for all group-wise comparisons, and *t*-tests were performed with the Python SciPy (1.1.0) [186] library to test for differences and generate statistics for downstream analyses. Any metabolite with *p* value < 0.05 was considered significantly regulated (up or down). Heatmaps were generated with hierarchical clustering performed on the imputed matrix values utilizing the R library Pheatmap (1.0.12) [187]. Volcano plots were generated utilizing the R library, Manhattanly (0.2.0). In order to adjust for significant covariate effects (as applicable) in the experimental design the R package, DESeq2 (1.24.0) [188] was used to test for significant differences. Data processing for this correction required the blank corrected matrix to be imputed with zeroes for non-detected values instead of the blank threshold to avoid false positives. This corrected matrix was then analyzed utilizing DESeq2 to calculate the adjusted *p* value in the covariate model. Final graphing for volcano plots was performed in Graphpad Prism v6.0.

### Design & statistical analysis

GraphPad Prism v6.0 was used for all statistical analysis and graphing. All data shown in the manuscript represents Mean ± Standard Error of the Mean (SEM). ANOVAs were the principal statistical test employed to control for the number of group comparisons being completed, as well as correct them when necessary. For comparisons that specifically involved *post hoc* comparisons or a specific comparison of just two groups, a *t* test was utilized. Cohen’s *d* was adapted for effect size estimation when evaluated. Significance was generally set at *p* < 0.05 per Fisher’s tables, tailed according to statistical-directionality guidelines and corrected for multiple-comparisons. To temper the potential influence of extraneous variance, we adapted unbiased, high-throughput, and analytical methodology where possible (e.g., TMT-LC/MS proteomics, metabolomics, and multiple flow cytometry experiments). This precluded the possibility extraneous variance arising from experimenter bias within these experiments. We also adapted a high-content approach that was concordant with principles of sampling theory. Specifically, we generated, observed, and analyzed as many units of analysis as possible. This resulted in hundreds of organoids being generated and randomly selected for experiments, that were each generated from multiple independent batches. Individual group numbers have been provided throughout the manuscript for clarity. Cellular quantifications were typically conducted in 5000 µm^2^ Regions of Interest (ROI) that typically extended 0–50 µm and 51–100 µm radially from ventricular zones. Flow cytometry data was analyzed and presented both as a global density %, normalized to total number of cellular singlet events for cell-cycle analysis and as fold-change for apoptosis and DNA damage analyses. Computational analyses were completed, and corrected for false discovery rates, as described above.

## Supplementary information

Supplementary Table 1. Global Analysis of TMT-LC:MS Proteomics by ANOVA

Supplementary Table 2. Global Pairwise Analysis of all TMT-LC:MS Quantifiable Proteins

Supplementary Table 3. Summary of Significant TMT-LC:MS Pairwise Comparisons

Supplementary Table 4. Distinct and Common Proteome Alterations Across Groups

## Data Availability

The MS-based proteomics data have been deposited in PeptideAtlas repository and are available with identifier PASS01187.
